# Chemoenzymatic synthesis of the cardenolide rhodexin A and its aglycone sarmentogenin

**DOI:** 10.3762/bjoc.21.204

**Published:** 2025-12-03

**Authors:** Fuzhen Song, Mengmeng Zheng, Dongkai Wang, Xudong Qu, Qianghui Zhou

**Affiliations:** 1 Engineering Research Center of Organosilicon Compounds & Materials (Ministry of Education), Hubei Key Lab on Organic and Polymeric OptoElectronic Materials, College of Chemistry and Molecular Sciences, The Institute for Advanced Studies, TaiKang Center for Life and Medical Sciences and State Key Laboratory of Metabolism and Regulation in Complex Organisms, Wuhan University, Wuhan, 430072, China; 2 School of Life Sciences, Shanghai University, Shanghai, 200444, China; 3 State Key Laboratory of Microbial Metabolism and School of Life Sciences and Biotechnology, Zhangjiang Institute for Advanced Study, Shanghai Jiao Tong University, Shanghai, 200240, China

**Keywords:** cardiac glycosides, C–H hydroxylation, chemoenzymatic synthesis, Mukaiyama hydration, protecting-group-free synthesis

## Abstract

Herein, we report a concise chemoenzymatic synthesis of the cardenolide rhodexin A in 9 steps and the first protecting-group-free synthesis of its aglycone sarmentogenin in 7 steps from 17-deoxycortisone. The synthesis features a scalable enzymatic C_14_–H α-hydroxylation, a Bestmann ylide-enabled one-step construction of the butenolide motif, a late stage Mukaiyama hydration, and a stereoselective C11 carbonyl reduction.

## Introduction

**Figure 1:**
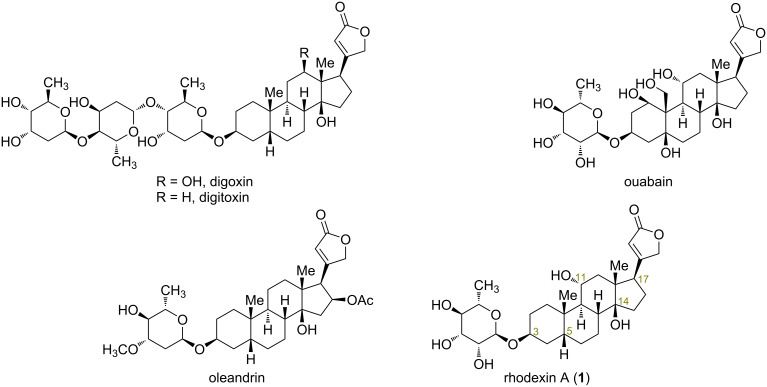
Representative CGs with promising biological activities.

Cardiac glycosides (CGs) are widely distributed natural products, generated by plants and amphibians [1]. Structurally, they are composed of an aglycone-steroidal moiety, an unsaturated lactone ring attached to the C17 position, and a glycosyl moiety in general. It is believed that CGs can increase cardiac contractility by inhibiting the sodium–potassium adenosine triphosphatase (Na^+^/K^+^ ATPase) of the plasma membrane [2]. The well-known CGs, such as digoxin, digitoxin, ouabain, and oleandrin have been used in clinical treatment for heart failure for a long time (Figure 1) [3–5]. The bioactivity of CGs is primarily determined by the lactone ring, with the sugar residue critically influencing their toxicological profile [6]. This is evident as the free aglycone facilitates absorption and metabolism, and specific moieties like rhamnose can enhance CG potency markedly by more than 25 times [1,7]. In addition, the -OH groups on the steroids’ structure also play an important role on their activity. However, these compounds are mainly isolated from plants or animals, which not only causes damage to the environment, but also greatly restricts further research for their pharmaceutical applications. Therefore, substantial synthetic efforts have been devoted towards the preparation of these valuable targets recently [8–17].

Rhodexin A was firstly isolated from the leaves and roots of the Japanese evergreen plant *Rohdea japonica* in 1951, and was also discovered in several other plants later [18]. Rhodexin A is the only natural CG that exhibits both cardiotonic activity and strong inhibitory activity on human leukemia K562 cells with an IC_50_ = 19 nM [19]. In addition, rhodexin A exhibits a strong antiproliferation activity due to the ability to inhibit the synthesis of hypoxia inducible factor 1 (HIF-1α) [20]. Thus, rhodexin A shows high value in medicinal research. Structurally, rhodexin A consists of two parts, the cardenolide aglycon – sarmentogenin and ʟ-rhamnose connected by the C3–O bond. In the steroidal skeleton, both the A/B and C/D rings are *cis* fused, which is different from common steroids. Besides, the steroidal skeleton is moderately oxidized at the C3, C11, and C14 positions. The introduction of the hydroxy groups and the glycoside in a stereocontrolled manner makes the synthesis of rhodexin A a challenging task. Currently, there are only a few reports about the synthesis of rhodexin A [20–22]. In 2011, the Jung group finished the first total synthesis of rhodexin A in 26 steps [20]. In this work, an elegant inverse-electron demand Diels–Alder (IEDDA) reaction was utilized to successfully construct the BCD tricyclic structure with the correct configuration of the four contiguous stereocenters in just one step. However, the requirement of a series of protecting group manipulations undermined the step-economy and overall synthetic efficiency of this route. Therefore, the development of more efficient syntheses of rhodexin A is of great significance.

**Scheme 1:**
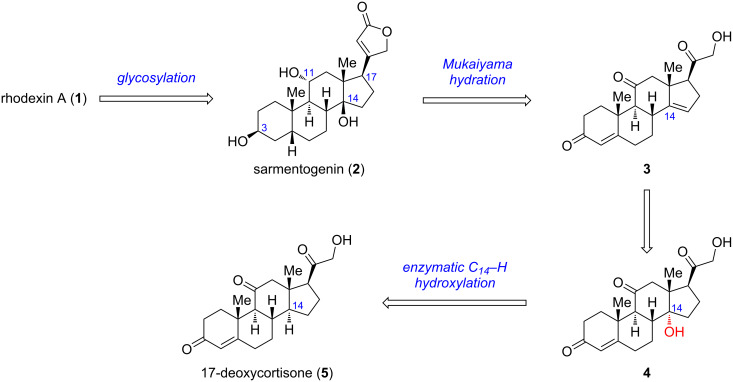
Retrosynthetic analysis of rhodexin A and sarmentogenin.

Recently, chemoenzymatic syntheses of steroids have made excellent progress, which can enormously shorten the synthetic routes and increase the overall efficiency [23–27]. Considering the common steroidal skeleton, we envisioned a chemoenzymatic strategy for the concise synthesis of rhodexin A and the retrosynthetic analysis is shown in Scheme 1. We envisaged rhodexin A (**1**) could be assembled from two fragments, sarmentogenin (**2**) and the ʟ-rhamnose donor, through a late-stage glycosylation. The aglycon **2** can be derived from the Δ14 olefin intermediate **3** via Mukaiyama hydration and several functional group transformations. In turn, **3** would be generated from the key C14α-hydroxylated intermediate **4** via an elimination process. For the synthesis of **4**, an enzymatic C14–H α-hydroxylation of 17-deoxycortisone (**5**) could be adopted, as described in our recent work [27]. Notably, **5** can be readily obtained from the inexpensive commercial steroid cortisone via a two-step process [27].

## Results and Discussion

Following the retrosynthetic analysis, we started the first step to prepare the C14α-hydroxylated steroidal intermediate **4** from 17-deoxycortisone (**5**). Delightfully, based on our previous study on enzymatic α-hydroxylation of C_14_–H of common steroids [27], compound **4** could be successfully obtained in 69% yield by using the biocatalyst CYP14A mutant. However, the maximum substrate loading was only 0.1 g/L, which was not sufficient for the enrichment of **4**. Thus, we changed our focus to another microorganism *Thamnostylum piriforme NBRC 6117*, which could act as whole cell biocatalyst to oxidize progesterone at the C14 position [28]. Fortunately, 17-deoxycortisone **5** could also be transformed into **4** in 65% yield after incubation in the *Thamnostylum piriforme NBRC 6117* liquid medium (Table 1, entry 1). Notably, a major C9 α-hydroxylated side product **4'** was also generated in 30% yield, which could be readily separated by column chromatography.

**Table 1:** Optimization of the fermentation conditions of the biocatalytic C_14_–H α-hydroxylation.

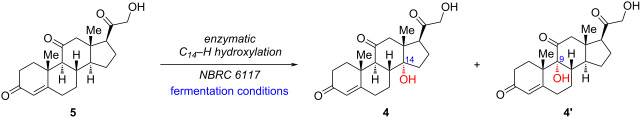					

Entry	Substrate loading (g/L)	Additive	Time (d)	Yield of **4** (%)^a^	Yield of **4'** (%)^a^

1	0.1	–	1	65	30
2	0.25	–	2	64	32
3	0.5	–	4	30	18
4	0.5	HP-β-CD	3	60	34
5	1.0	HP-β-CD	4	52	26
**6**	**1.0**	**HP-β-CD** **tween 80**	**4**	**64**	**30**
7	2.0	HP-β-CD tween 80	6	28	15

^a^Isolated yields were reported.

Based on the above preliminary results, we subsequently performed extensive optimization of fermentation conditions for a scalable synthesis of **4**. As shown in Table 1, when the substrate loading was increased to 0.25 g/L, a much longer reaction time (2 days) was required to allow complete conversion, affording **4** in 64% yield (Table 1, entry 2). However, at a higher concentration (0.5 g/L), a significant amount of starting compound **5** remained unconverted even after 4 days, and only 30% of **4** was isolated (Table 1, entry 3). To our delight, upon adding 2-hydroxypropyl-β-cyclodextrin (HP-β-CD) [29] as the solubilizer to the fermentation broth, a complete conversion of **5** was achieved within 3 days to afford **4** in 60% yield (Table 1, entry 4). When the substrate loading was further increased to 1.0 g/L in the presence of HP-β-CD, there was a small amount of starting material remained after 4 days of fermentation (Table 1, entry 5). Delightfully, after adding 1% Tween 80 to the above fermentation broth, a complete conversion was observed in 4 days, and compound **4** was obtained in 64% yield (Table 1, entry 6). However, further increasing the substrate concentration to 2.0 g/L resulted in a much lower conversion rate owing to inhibited microbial growth (Table 1, entry 7). Therefore, the conditions from Table 1, entry 6 were identified as the optimized fermentation conditions that secured the gram-scale synthesis of **4** effortlessly.

**Scheme 2:**
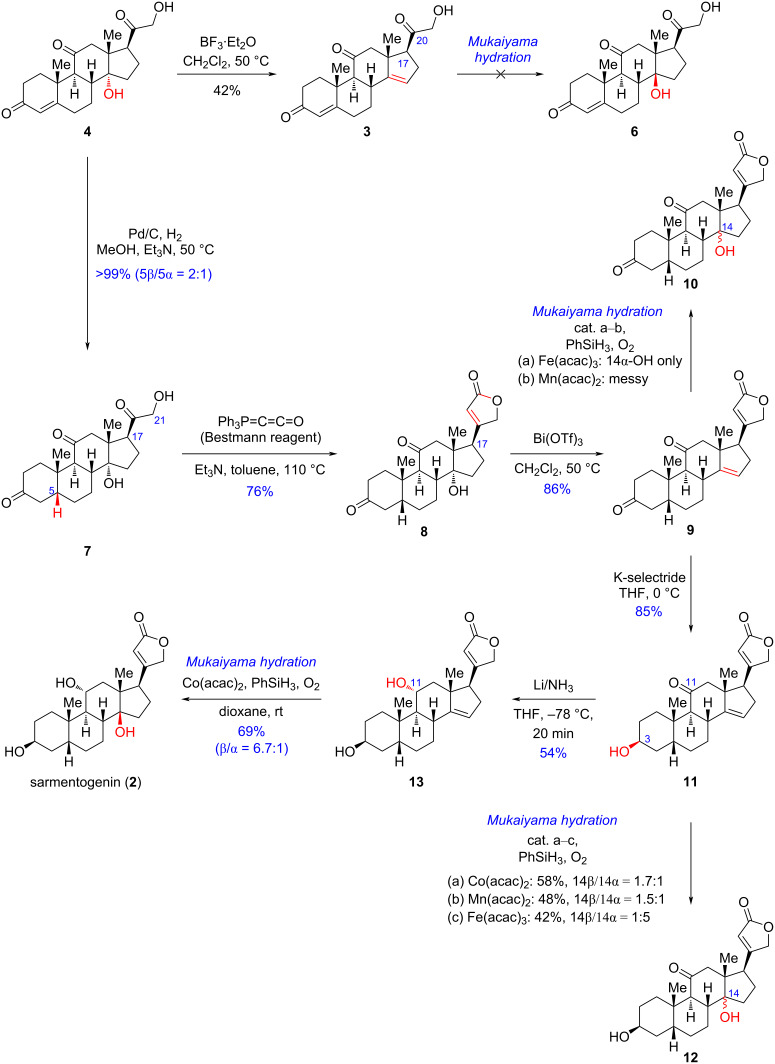
Chemoenzymatic synthesis of sarmentogenin (**2**).

With compound **4** in sufficient quantities at hands, we next focused on transforming it into the pivotal C14 β-hydroxylated steroidal intermediate. As shown in Scheme 2, a BF_3_·Et_2_O-promoted elimination afforded the 14-olefinated intermediate **3** in a moderate yield. However, the following Mukaiyama hydration to introduce the C14 β-hydroxy group was unsuccessful. Owing to the reactive C17 side chain including an α-hydroxycarbonyl group, a set of side reactions (e.g., reduction of the C20 carbonyl, hydrogenation of Δ^4^ and Δ^14^ double bonds, etc.) occurred under the Mukaiyama hydration conditions [30–31]. Therefore, it was necessary to alter the side chain before installing the C14 β-OH group.

The revised synthetic route is described in Scheme 2. At first, **4** was subjected to a Pd/C-catalyzed hydrogenation to afford the desired A/B-*cis* fused intermediate **7** along with its C5 epimer as a 2:1 separable mixture in a quantitative yield. By treating **7** with the Bestmann ylide reagent [20], the key intermediate **8** bearing a butenolide motif was obtained in 76% yield. Next, with the aid of the strong Lewis acid Bi(OTf)_3_, the regioselective elimination of **8** was achieved to produce the Δ^14^ olefin intermediate **9** in 86% yield. Afterwards, we evaluated the reactivity of **9** towards typical Mukaiyama hydration conditions [30–32] to install the C14 β-hydroxy group. However, only a trace amount of the undesired C14α-hydroxylated product **10** was obtained. Additional optimizations regarding the transition-metal catalyst, hydrogen source and solvent all failed to improve the results. Therefore, we decided to perform the Mukaiyama hydration on advanced intermediates. Next, a K-selectride-promoted chemo- and stereoselective reduction of the C3 carbonyl of **9** was realized to solely deliver **11** in 85% yield [33]. Then, **11** was subjected to Mukaiyama hydration conditions. Under the Fe(acac)_3_-catalyzed anaerobic conditions [30], **11** was transformed into the C14 hydroxylated intermediate **12** as epimeric mixture in 42% yield and dr = 1:5, among which the undesired 14α-hydroxy epimer was the major component. Interestingly, the use of Co(acac)_2_ or Mn(acac)_2_ as the catalyst [31–32] instead of Fe(acac)_3_ could reverse the diastereomeric ratio of the C14-hydroxylated products to dr = 1.5–1.7:1 and increased the yield to 48–58% as well. Nevertheless, the unsatisfactory diastereoselectivity urged us to explore a late-stage Mukaiyama hydration. To this end, we next performed the thermodynamic C11-carbonyl reduction by dissolved lithium metal in liquid NH_3_ solution, and the C11 α-hydroxylated intermediate **13** was obtained as a single diastereoisomer in 54% yield. Lastly, as expected, a Co(acac)_2_-catalyzed Mukaiyama hydration of **13** afforded the desired natural product sarmentogenin (**2**) in 69% yield with high diastereoselectivity (dr = 14β/14α = 6.7:1), and the minor component 14-*epi*-sarmentogenin could be readily separated. Notably, our work represents the first protecting-group-free synthesis [34–35] of sarmentogenin in just 7 steps from 17-deoxycortisone.

**Scheme 3:**
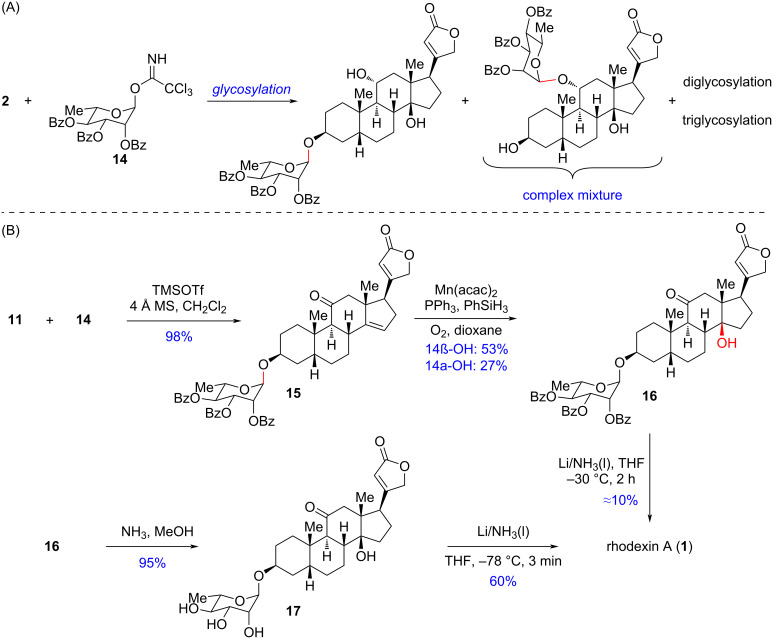
Synthesis of rhodexin A.

With the aglycone sarmentogenin (**2**) in hand, we first tried the synthesis of rhodexin A through direct glycosylation of **2** by the ʟ-rhamnose donor 2,3,4-tri-*O*-benzoyl-α-ʟ-rhamnopyranosyl trichloroacetimidate (**14**). However, the selective glycosylation at the C3-hydroxy group of **2** was a formidable challenge since competitive glycosylations of the other hydroxy groups of **2** could not be avoided, resulting in a complex mixture including mono-, di-, and triglycosylated products (Scheme 3A). Thus, both C11–OH and C14–OH needed to be masked before glycosylation. Based on this thought, we selected intermediate **11** as a suitable substrate for glycosylation. To our delight, as shown in Scheme 3B, with TMSOTf as the promoter, the glycosylation between **11** and **14** proceeded smoothly in a stereospecific manner, delivering the key intermediate **15** in 98% yield. Subsequently, treating **15** under Mn(acac)_2_-catalyzed Mukaiyama hydration conditions yielded the key C14 β-hydroxylated intermediate **16** in 53% yield, accompanied by the separable C14 α-OH epimer in 27% yield. Later on, we assumed to achieve the deprotection of the sugar motif and the reduction of the C11 carbonyl of **16** simultaneously under Li–NH_3_(l) conditions, which would directly afford the final natural product rhodexin A in one step. Just as expected, when directly subjecting **16** to the abovementioned conditions, rhodexin A was indeed afforded albeit in only 10% yield. The required longer reaction time and elevated temperature to achieve deprotection of the sugar motif resulted in an over-reduction of the butenolide motif. The low efficiency of this reaction prompted us to pursue an alternative two-step approach. First, we performed the deprotection to remove all the Bz groups of **16** by NH_3_/MeOH, furnishing the saccharide **17** in 95% yield. Eventually, the quick stereospecific C11 carbonyl reduction by Li–NH_3_(l) was accomplished in just 3 minutes to afford rhodexin A in 60% isolated yield. The synthetic sample exhibited identical NMR spectroscopic data to the literature precedent [20], which confirmed the completion of the chemoenzymatic synthesis of rhodexin A in 9 steps from 17-deoxycortisone.

## Conclusion

In summary, we have completed a concise chemoenzymatic synthesis of cardenolide rhodexin A in 9 steps and the first protecting-group-free synthesis of its aglycone sarmentogenin (**2**) in 7 steps from 17-deoxycortisone. The key steps include a scalable enzymatic C_14_–H α-hydroxylation, a Bestmann ylide-enabled one-step construction of the butenolide motif, a late-stage Mukaiyama hydration, and a stereoselective C11 carbonyl reduction. We believe this chemoenzymatic synthetic strategy will inspire future endeavors towards the practical synthesis of complex steroids and other bioactive natural products.

## Supporting Information

File 1 Experimental details and spectral data for all new compounds.

## Data Availability

All data that supports the findings of this study is available in the published article and/or the supporting information of this article.
