# Supplementary material for: Chemoenzymatic synthesis of the cardenolide rhodexin A and its aglycone sarmentogenin
Source: Beilstein J Org Chem. 2025 Dec 3;21:2637–44. doi: 10.3762/bjoc.21.204 (PMC12684428; doi:10.3762/bjoc.21.204)
Supplement: File 1 — Experimental details and spectral data for all new compounds. [file Beilstein_J_Org_Chem-21-2637-s001.pdf]

BEILSTEIN JOURNAL OF ORGANIC CHEMISTRY

Supporting Information

for

Chemoenzymatic synthesis of the cardenolide rhodexin A and its aglycone sarmentogenin

Fuzhen Song, Mengmeng Zheng, Dongkai Wang, Xudong Qu and Qianghui Zhou

Beilstein J. Org. Chem. **2025,** *21,* 2637–2644. doi:10.3762/bjoc.21.204

Experimental details and spectral data for all new compounds

License and Terms: This is a supporting information file under the terms of the Creative Commons Attribution License (https://creativecommons.org/licenses/by/4.0). Please note that the reuse, redistribution and reproduction in particular requires that the author(s) and source are credited and that individual graphics may be subject to special legal provisions.
The license is subject to the *Beilstein Journal of Organic Chemistry* terms and conditions: (https://www.beilstein-journals.org/bjoc/terms)

Table of contents

1. General information S1
2. Experimental details S1
3. Data comparison S12
4. References S16
5. Copies of NMR spectra S17

1. General information

All solvents were dried with a JC Meyer Solvent Drying System unless otherwise specified. Most reagents were purchased from commercial sources and used without further purification, unless otherwise stated. Reactions were monitored by TLC carried out on 0.2 mm commercial silica gel plates, using UV light as the visualizing agent or a solution of phosphomolybdic acid in ethanol or an acidic solution of H_2SO_4 in methanol and heat as the developing agent. ^{1}H NMR and ^{13}C NMR spectra were recorded with a Bruker DMx 400 spectrometer (400 MHz; ^{1}H at 400 MHz, ^{13}C at 100 MHz, ^{19}F at 376 MHz) and Bruker a AVANCE NEO 600 spectrometer (600 MHz; ^{1}H at 600 MHz, ^{13}C at 151 MHz). Chemical shifts are reported in parts per million (ppm, δ) with residual solvent resonances as the internal standard ($CDCl_3$ at δ 7.26, δ 77.16 or CD_3OD at δ 3.31, δ 49.0). Tabulated ^{1}H NMR data are reported as s = singlet, d = doublet, t = triplet, q = quartet, qn = quintet, sext = sextet, m = multiplet, ovrlp = overlap, and coupling constants in Hz. Coupling constants are reported in hertz (Hz). Data for ^{1}H NMR spectra are reported as follows: chemical shift (ppm, referenced to protium), multiplicity (standard abbreviations), coupling constant (Hz), and integration. Microwave reactions were carried out with a @ 2011 Biotage Sweden AB Synthesizer. Optical rotation was determined using a Perkin Elmer 343 polarimeter. High resolution mass spectra (HRMS) were recorded on DIONEx UltiMate3000 & Bruker Compact TOF mass spectrometer.

2. Experimental details

General procedure for *T. piriforme* mycelium: Firstly, the preserved *T. piriforme* strain is inoculated onto a fresh PDA solid plate medium. It is then incubated at 28 °C for several days until the mycelium fully covers the plate, yielding an activated culture. To scale up the culture, mycelial plugs are taken from the activated plate and inoculated into a 250 mL flask containing100 mL liquid medium (standard protocol: yeast extract: 25 g/L, glucose: 20 g/L). This is then shake-cultured for two days at 28 °C (200 rpm) to obtain mycelial pellets.

a) Synthesis of 14-hydroxy-17-deoxycortisone (4)

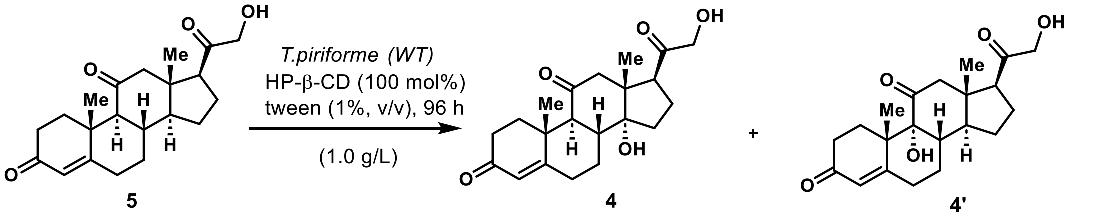

Firstly, *T. piriforme* pellets were obtained following the above general procedure. Then the pellets were transferred to 2 L liquid medium (yeast extract: 25 g/L, glucose: 20 g/L, HP-β-CD: 100 mol %, tween 80: 1 vol %), and grown for two days at 28 °C (200 rpm). A solution of compound **5** (2.0 g, 5.8 mmol) in *N*,*N*-dimethylformamide (20 mL) was then injected it into the aforementioned culture medium using a syringe. The reaction was continued for 4 days, and the same volume of ethyl acetate or dichloromethane was added for repeated extraction. The obtained organic phase was concentrated under vacuum, and then separated by silica gel column chromatography (CH_2Cl_2/EtOAc 1:1) to obtain the target hydroxylation product **4** (1.3 g, 64% yield) and C9α-hydroxylated product **4'** (610.0 mg, 30% yield).

Compound 4

^{1}H NMR (400 MHz, $CDCl_3$) δ 5.72 (s, 1H), 4.23–4.06 (m, 2H), 3.38 (t, *J* = 9.0 Hz, 1H), 3.08 (d, *J* = 12.5 Hz, 1H), 2.84 (dt, *J* = 13.6, 4.3 Hz, 1H), 2.50–2.22 (m, 9H), 2.10–1.95 (m, 1H), 1.94–1.77 (m, 3H), 1.76–1.53 (m, 3H), 1.39 (s, 3H), 0.74 (s, 3H);

^{13}C NMR (101 MHz, $CDCl_3$) δ 210.2, 208.6, 200.1, 168.4, 124.8, 83.8, 69.5, 57.0, 53.6, 51.8, 50.1, 40.2, 38.3, 35.0, 33.9, 33.2, 32.2, 27.3, 22.2, 18.7, 17.2.

The NMR data were consistent with the reported literature.[1]

Compound 4'

Physical state: white solid;

Melting point: 210–212 °C;

$[\alpha]_D$ = +111.1 (c = 0.3, $CHCl_3$);

R_f = 0.1 (2:1 CH_2Cl_2: EtOAc);

HRMS (ESI–TOF): calc'd for $C_{21}H_{29}O_5^+$ [M+H$^+$] 361.2009, found 361.2010.

^{1}H NMR (400 MHz, $CDCl_3$) δ 5.86 (d, J = 2.0 Hz, 1H), 4.24 – 4.10 (m, 2H), 3.18 (t, J = 4.8 Hz, 1H), 3.06 (d, J = 11.8 Hz, 1H), 2.73 (t, J = 9.4 Hz, 1H), 2.55 – 2.42 (m, 3H), 2.39 – 2.14 (m, 7H), 2.13 – 2.03 (m, 1H), 1.99 – 1.78 (m, 2H), 1.69 (ddd, J = 16.1, 9.0, 4.6 Hz, 2H), 1.48 (s, 3H), 1.41 (td, J = 11.9, 6.1 Hz, 1H), 0.67 (s, 3H);

^{13}C NMR (101 MHz, $CDCl_3$) δ 209.3, 207.1, 199.3, 166.5, 127.7, 79.5, 69.4, 57.6, 52.1, 48.9, 47.3, 43.8, 38.8, 33.9, 31.5, 28.3, 24.6, 23.9, 23.5, 19.1, 14.1.

b) Synthesis of (5*R*,8*R*,9*S*,10*S*,13*R*,14*R*,17S)-14-hydroxy-17-(2-hydroxyacetyl)-10,13-dimethyltetradecahydro-3*H*-cyclopenta[*a*]phenanthrene-3,11(2*H*)-dione (7)

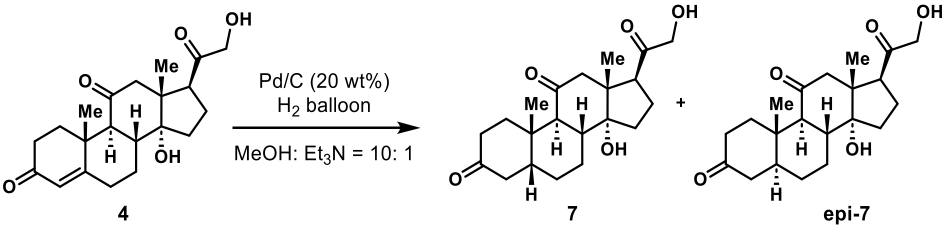

To a dry flask was added compound **4** (600.0 mg, 1.67 mmol) and 5% Pd/C (120 mg, 20 wt %). MeOH (24 mL) was added under Ar. Then, the solution was bubbled with H_2 for 10 min, followed by the addition of Et_3N (2.4 mL, 10 vol %). The resulting mixture was stirred at room temperature for overnight. After completion, the mixture was filtered over a pad of Celite and rinsed with EtOAc (50 mL), then concentrated under vacuum. The crude product was purified by silica gel column chromatography (CH_2Cl_2/EtOAc 1:2) to afford compound **7** (400.0 mg, 67%) and compound ***epi*-7** (186.0 mg, 31%) as white solids.

Compound 7

Physical state: white solid;

Melting point: 196–198 °C;

Rf = 0.2 (1:2 CH_2Cl_2: EtOAc);

[α]D = +85.9 (c = 0.26, $CHCl_3$);

HRMS (ESI-TOF): calc'd for $C_{21}H_{30}O_5Na^+$, [M+Na$^+$] 385.1985; found, 385.1987;

^{1}H NMR (400 MHz, $CDCl_3$) δ 4.25 – 4.08 (m, 2H), 3.38 (t, *J* = 9.0 Hz, 1H), 3.20 (t, *J* = 4.7 Hz, 1H), 3.11 (d, *J* = 12.5 Hz, 1H), 3.01 (d, *J* = 11.5 Hz, 1H), 2.85 (dt, *J* = 14.4, 4.6 Hz, 1H), 2.58 (dd, *J* = 15.4, 13.7 Hz, 1H), 2.38 (m, 1H), 2.29 – 2.18 (m, 3H), 2.14 (td, *J* = 11.9, 3.9 Hz, 1H), 2.09 – 2.00 (m, 2H), 2.00 – 1.82 (m, 2H), 1.81 – 1.64 (m, 4H), 1.50 (m, 2H), 1.40 – 1.30 (m, 1H), 1.21 (s, 3H), 0.71 (s, 3H);

^{13}C NMR (101 MHz, $CDCl_3$) δ 212.8, 210.3, 209.7, 84.3, 69.5, 53.7, 51.8, 50.3, 46.1, 44.8, 42.4, 39.9, 37.6, 36.6, 34.6, 33.3, 25.9, 22.2, 22.1, 21.3, 18.9.

Compound *epi*-7

Physical state: white solid;

Melting point: 155–157 °C;

Rf = 0.25 (1:2 CH_2Cl_2: EtOAc);

[α]D = +84.0 (c = 0.53, $CHCl_3$);

HRMS (ESI-TOF): calc'd for $C_{21}H_{31}O_5^+$, [M+H$^+$] 363.2166; found, 363.2157;

^{1}H NMR (400 MHz, $CDCl_3$) δ 4.15 (t, *J* = 4.3 Hz, 2H), 3.38 (t, *J* = 9.0 Hz, 1H), 3.20 (t, *J* = 4.6 Hz, 1H), 3.07 (d, *J* = 12.3 Hz, 1H), 2.88 (ddd, *J* = 13.4, 6.6, 2.4 Hz, 1H), 2.51 – 2.17 (m, 7H), 2.16 – 1.94 (m, 3H), 1.85 (m, 1H), 1.77 – 1.60 (m, 5H), 1.50 (m, 1H), 1.33 (m, 1H), 1.19 (s, 3H), 0.70 (s, 3H);

^{13}C NMR (101 MHz, $CDCl_3$) δ 211.5, 210.3, 209.6, 84.1, 69.5, 57.7, 53.7, 51.8, 50.1, 46.7, 44.4, 39.8, 38.1, 37.2, 35.4, 33.3, 28.0, 27.2, 22.2, 18.7, 11.0

c) **Synthesis of (5*R*,8*R*,9*S*,10*S*,13*R*,14*R*,17*R*)-14-hydroxy-10,13-dimethyl-17-(5-oxo-2,5-dihydrofuran-3-yl)tetradecahydro-3*H*-cyclopenta[*a*]phenanthrene-3,11(2*H*)-dione (8)**

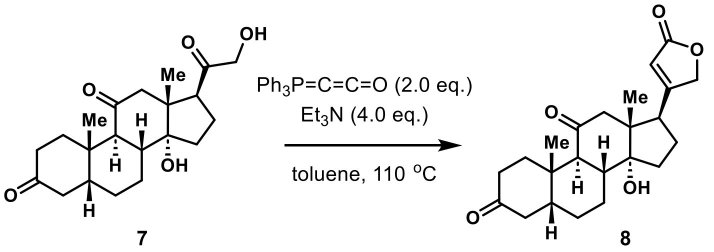

To a 100 mL dry flask was added compound **7** (350 mg, 0.97 mmol) and $Ph_3P=C=C=O$ (586 mg, 1.94 mmol). Toluene (35 mL) was added under Ar. Then, Et_3N (542 µL, 3.87 mmol) was added dropwise. The resulting mixture was warmed to 110 °C and stirred for 3 h. The mixture was cooled to room temperature and concentrated under vacuum. The crude product was purified by silica gel column chromatography (CH_2Cl_2/EtOAc 1:1) to afford compound **8** (284.0 mg, 76%) as a white solid.

Physical state: white solid;

Melting point: 250–252 °C;

R_f = 0.3 (1:2 CH_2Cl_2: EtOAc);

$[\alpha]_D$ = +24.9 (c = 0.24, $CHCl_3$);

HRMS (ESI-TOF): calc'd for $C_{23}H_{31}O_5^+$, [M+H$^+$] 387.2166; found, 387.2169;

^{1}H NMR (400 MHz, $CDCl_3$) δ 5.89 (d, J = 1.8 Hz, 1H), 4.71 (qd, J = 17.5, 1.8 Hz, 2H), 3.41 (t, J = 8.6 Hz, 1H), 3.03 (dd, J = 11.9, 9.0 Hz, 2H), 2.85 (dt, J = 14.4, 4.6 Hz, 1H), 2.58 (dd, J = 15.4, 13.7 Hz, 1H), 2.33 – 2.10 (m, 4H), 2.10 – 2.01 (m, 2H), 2.01 – 1.89 (m, 4H), 1.82 – 1.67 (m, 3H), 1.62 (s, 1H), 1.53 – 1.44 (m, 1H), 1.41 – 1.31 (m, 1H), 1.22 (s, 3H), 0.70 (s, 3H);

^{13}C NMR (101 MHz, $CDCl_3$) δ 212.7, 209.5, 173.7, 170.3, 117.3, 83.7, 73.4, 51.9, 49.8, 46.2, 45.0, 44.8, 42.4, 40.4, 37.6, 36.6, 34.6, 33.4, 25.8, 25.1, 22.2, 21.1, 18.4.

d) **Synthesis of (5*R*,8*R*,9*S*,10*S*,13*R*,17*S*)-10,13-dimethyl-17-(5-oxo-2,5-dihydrofuran-3-yl)-1,4,5,6,7,8,9,10,12,13,16,17-dodecahydro-3*H*-cyclopenta[*a*]phenanthrene-3,11(2*H*)-dione (9)**

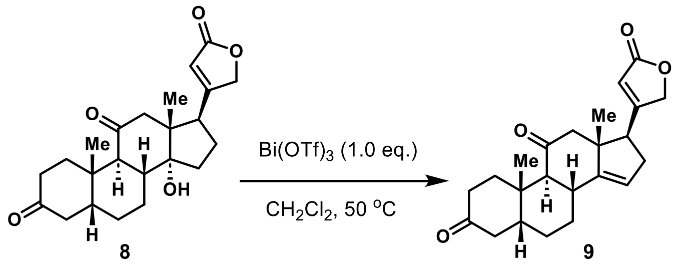

To a solution of compound **8** (200 mg, 0.52 mmol) in CH_2Cl_2 (10 mL), $Bi(OTf)_3$ (320 mg, 0.52 mmol) was added under Ar. The resulting mixture was heated to 50 °C and stirred for 2 h. After completion, the reaction was quenched by the addition of saturated $NaHCO_3$ solution (20 mL). The mixture was extracted by EtOAc (3 × 20 mL) and washed with saturated NaCl solution (3 × 25 mL). The organic layer was dried over anhydrous Na_2SO_4, filtered and concentrated under vacuum. The crude product was purified by silica gel column chromatography (CH_2Cl_2/EtOAc 5:1) to afford compound **9** (190.0 mg, 86%) as a white solid.

Physical state: white solid;
Melting point: 270–272 °C;
Rf = 0.6 (1:2 CH_2Cl_2: EtOAc);
[α]D = +4.2 (c = 0.14, $CHCl_3$);
HRMS (ESI-TOF): calc'd for $C_{23}H_{29}O_4^+$, [M+H$^+$] 369.2060; found, 369.2060;
^{1}H NMR (400 MHz, $CDCl_3$) δ 5.94 (s, 1H), 5.55 (d, *J* = 2.7 Hz, 1H), 5.01 – 4.46 (m, 2H), 3.04 (t, *J* = 9.1 Hz, 1H), 2.74 – 2.35 (m, 8H), 2.27 – 1.89 (m, 5H), 1.85 – 1.63 (m, 2H), 1.59 – 1.47 (m, 1H), 1.39 (dq, *J* = 12.2, 2.5 Hz, 1H), 1.30 (s, 3H), 0.85 (s, 3H);
^{13}C NMR (101 MHz, $CDCl_3$) δ 211.9, 208.0, 173.4, 168.5, 149.6, 120.1, 117.3, 73.1, 57.0, 52.6, 51.7, 50.8, 44.7, 42.0, 37.5, 36.2, 35.2, 34.6, 34.1, 25.6, 24.1, 22.2, 20.0.

e) Synthesis of 4-((3*S*,5*R*,8*R*,9*S*,10*S*,13*R*,17*S*)-3-hydroxy-10,13-dimethyl-11-oxo-2,3,4,5,6,7,8,9,10,11,12,13,16,17-tetradecahydro-1*H*-cyclopenta[*a*]phenanthren-17-yl)furan-2(5*H*)-one (11)

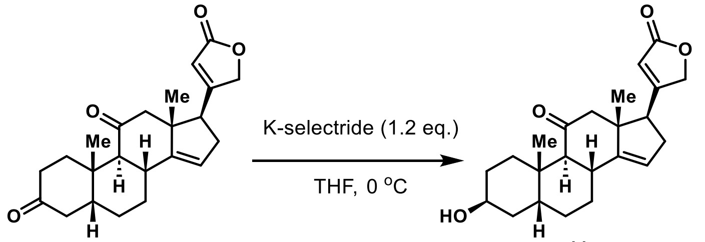

The compound **9** (100 mg, 0.27 mmol) was dissolved in THF (25 mL) under Ar. After cooling to 0 °C, a 1 M solution of K-selectride in THF (324 μL, 0.32 mmol) was added dropwise. The resulting mixture was stirred at the same temperature for 3 hours and quenched by the addition of saturated NH_4Cl solution (10 mL). The mixture was extracted by EtOAc (25 mL) and washed with saturated NaCl solution (3 × 10 mL). The organic layer was dried over anhydrous Na_2SO_4, filtered and concentrated under vacuum. The crude product was purified by silica gel column chromatography (CH_2Cl_2/EtOAc 2:1) to afford compound **11** (85.0 mg, 85%) as a white solid.
Physical state: white solid;
Melting point: 66–68 °C;
Rf = 0.6 (1:3 CH_2Cl_2: EtOAc);
[α]D = +115.7 (c = 0.13, $CHCl_3$);
HRMS (ESI-TOF): calc'd for $C_{23}H_{31}O_4^+$, [M+H$^+$] 371.2217; found, 371.2214;
^{1}H NMR (400 MHz, $CDCl_3$) δ 5.92 (d, *J* = 1.6 Hz, 1H), 5.49 (d, *J* = 2.5 Hz, 1H), 4.81 – 4.66 (m, 2H), 4.09 (d, *J* = 2.5 Hz, 1H), 3.05 – 2.96 (m, 1H), 2.60 – 2.35 (m, 6H), 2.11 – 1.93 (m, 2H), 1.91 – 1.78 (m, 2H), 1.76 – 1.57 (m, 3H), 1.56 – 1.36 (m, 5H), 1.25 (s, 3H), 0.82 (s, 3H);
^{13}C NMR (101 MHz, $CDCl_3$) δ 208.9, 173.6, 169.0, 150.6, 119.6, 117.3, 73.3, 66.5, 57.4, 52.8, 51.9, 50.2, 37.0, 35.5, 35.0, 34.2, 33.4, 29.1, 28.6, 25.8, 24.7, 23.5, 20.0.

f) Synthesis of 4-((3*S*,5*R*,8*R*,9*S*,10*S*,13*R*,17*R*)-3,14-dihydroxy-10,13-dimethyl-11-oxohexadecahydro-1*H*-cyclopenta[*a*]phenanthren-17-yl)furan-2(5*H*)-one (12)

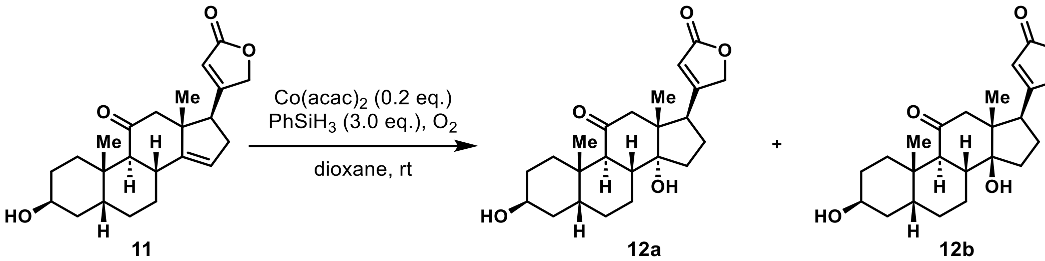

11 12a 12b

To a 25 mL dry flask was added compound **11** (50 mg, 0.14 mmol) and Co(acac)$_2$ (6.8 mg, 0.027 mmol). Then anhydrous 1,4-dioxane (5 mL) was added under O_2 atmosphere. After being stirred for 5 min, $PhSiH_3$ (50 µL, 0.4 mmol) was added dropwise. The reaction was carried out at room tempeature for 12 h. Saturated $Na_2S_2O_3$ solution (10 mL) was added to the mixture and stirred for 30 min. The mixture was extracted by EtOAc (10 mL) and washed with saturated NaCl solution (3 × 10 mL). The organic layer was dried over anhydrous Na_2SO_4, filtered and concentrated under vacuum. The crude product was purified by silica gel column chromatography (CH_2Cl_2/EtOAc 1:1) to afford compound **12a** (11.0 mg, 21%) and **12b** (19.0 mg, 37%) as white solids.

Compound 12a

Physical state: white solid;

Melting point: 199–201 °C;

***R*$_f$** = 0.3 (1:3 CH_2Cl_2: EtOAc);

[α]$_D$ = +26.4, (c = 0.11, $CHCl_3$);

HRMS (ESI-TOF): calc'd for $C_{23}H_{33}O_5^+$, [M+H$^+$] 389.2323; found, 389.2319;

^{1}H NMR (400 MHz, CDCl$_3$) δ 5.88 (d, *J* = 1.7 Hz, 1H), 4.75 (dd, *J* = 17.4, 1.8 Hz, 1H), 4.70 – 4.61 (m, 1H), 4.10 (t, *J* = 3.0 Hz, 1H), 3.39 (t, *J* = 8.6 Hz, 1H), 3.00 (d, *J* = 12.1 Hz, 1H), 2.91 (d, *J* = 11.5 Hz, 1H), 2.43 – 2.35 (m, 1H), 2.31 – 2.16 (m, 1H), 2.10 (td, *J* = 12.0, 4.0 Hz, 1H), 2.02 – 1.80 (m, 5H), 1.74 – 1.59 (m, 3H), 1.54 – 1.34 (m, 7H), 1.30 (d, *J* = 2.8 Hz, 1H), 1.18 (s, 3H), 0.67 (s, 3H);

^{13}C NMR (151 MHz, CDCl$_3$) δ 210.0, 173.6, 170.4, 117.2, 84.0, 73.4, 66.6, 51.8, 50.0, 45.1, 45.0, 40.5, 37.2, 35.1, 33.7, 33.6, 29.3, 28.7, 25.9, 25.2, 23.4, 21.5, 18.4.

Compound 12b

Physical state: white solid;

Melting point: 240–242 °C;

***R*$_f$** = 0.3 (1:3 CH_2Cl_2: EtOAc);

[α]$_D$ = –15.0 (c = 0.12, $CHCl_3$);

HRMS (ESI-TOF): calc'd for $C_{23}H_{33}O_5^+$, [M+H$^+$] 389.2323; found, 389.2320;

^{1}H NMR (400 MHz, CDCl$_3$) δ 5.91 (s, 1H), 4.81 (dd, *J* = 3.8, 1.9 Hz, 2H), 4.13 (d, *J* = 3.7 Hz, 1H), 2.58 (t, *J* = 7.8 Hz, 1H), 2.37 (dd, *J* = 14.2, 12.6 Hz, 2H), 2.23 (d, *J* = 13.1 Hz, 1H), 2.20 – 2.12 (m, 1H), 2.09 – 1.90 (m, 5H), 1.85 – 1.74 (m, 4H), 1.72 – 1.56 (m, 2H), 1.48 – 1.29 (m, 6H), 1.12 (s, 3H), 0.89 (s, 3H);

^{13}C NMR (151 MHz, CDCl$_3$) δ 211.5, 173.9, 170.6, 118.1, 83.8, 73.3, 66.5, 54.4, 51.6, 49.2, 47.4, 43.1, 36.8, 36.6, 34.5, 33.7, 29.5, 28.6, 26.5, 26.5, 24.0, 21.6, 18.7.

g) Synthesis of 4-((3*S*,5*R*,8*R*,9*S*,10*S*,11*R*,13*R*,17*S*)-3,11-dihydroxy-10,13-dimethyl-2,3,4,5,6,7,8,9,10,11,12,13,16,17-tetradecahydro-1*H*-cyclopenta[*a*]phenanthren-17-yl)furan-2(5*H*)-one (13)

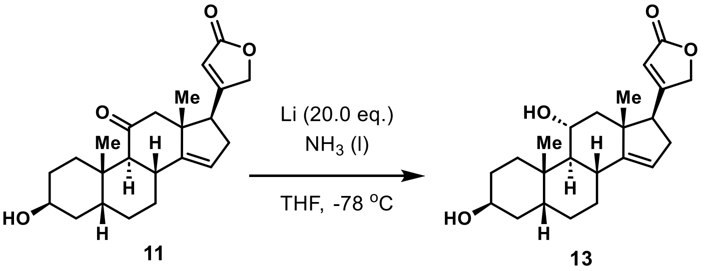

To a flask equipped with a dry-ice condenser containing ammonia at −78 °C was added lithium wire (14 mg, 2.0 mmol). After 10 minutes, compound **11** (35 mg, 0.1 mmol) in tetrahydrofuran (200 μL) was introduced to the blue ammonia solution at −78 °C. The reaction was allowed to stir for 20 min and then quenched with dropwise addition of a cosolvent THF/*t*-BuOH 10:1 (v/v, 3 mL), followed by saturated aq. NH_4Cl (5 mL). After warming to room temperature, the aqueous layer was extracted with EtOAc (3 × 5 mL) and washed with brine (10 mL). The organic layer was dried over anhydrous Na_2SO_4, filtered and concentrated under vacuum. The crude product was purified by silica gel column chromatography (CH_2Cl_2/EtOAc 1:1) to afford compound **13** (18.0 mg, 54%) as a white solid.

Physical state: white solid;
Melting point: 75–77 °C;
R_f = 0.1 (1:1 CH_2Cl_2: EtOAc);
$[\alpha]_D$ = -12.1 (c = 0.40, $CHCl_3$);
HRMS (ESI-TOF): calc'd for $C_{23}H_{33}O_4^+$ [M+H$^+$] 373.2373, found 373.2374;
^{1}H NMR (600 MHz, $CDCl_3$) δ 5.90 (d, J = 1.7 Hz, 1H), 5.28 (d, J = 2.4 Hz, 1H), 4.80 (dd, J = 17.5, 1.8 Hz, 1H), 4.75 – 4.69 (m, 1H), 4.12 (q, J = 4.3, 3.8 Hz, 1H), 3.92 (td, J = 10.4, 4.1 Hz, 1H), 2.84 (t, J = 9.2 Hz, 1H), 2.53 – 2.42 (m, 2H), 2.35 (dt, J = 13.9, 3.2 Hz, 1H), 2.10 – 2.02 (m, 2H), 1.93 (tt, J = 13.8, 4.0 Hz, 2H), 1.79 – 1.66 (m, 3H), 1.61 – 1.46 (m, 6H), 1.38 – 1.32 (m, 1H), 1.29 – 1.24 (m, 2H), 1.12 (s, 3H), 0.84 (s, 3H);
^{13}C NMR (151 MHz, $CDCl_3$) δ 174.0, 170.1, 152.8, 117.6, 116.7, 73.6, 69.8, 67.1, 52.7, 52.3, 48.9, 45.6, 38.3, 36.7, 33.9, 33.8, 33.5, 32.9, 28.9, 26.4, 23.7, 23.6, 19.5.

h) Synthesis of sarmentogenin (2)

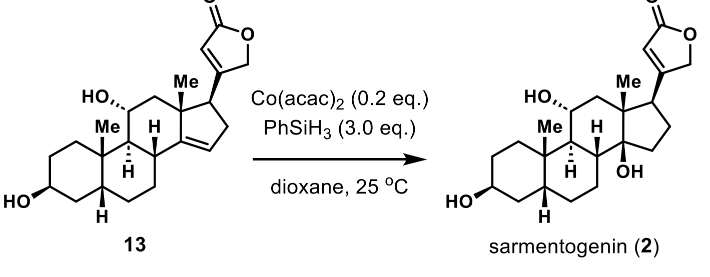

To a 25 mL dry flask was added compound **13** (7.5 mg, 0.02 mmol) and $Co(acac)_2$ (1.0 mg, 0.004 mmol). Then anhydrous 1,4-dioxane (5 mL) was added under O_2 atmosphere. After being stirred for 10 min, $PhSiH_3$ (7.4 μL, 0.06 mmol) was added dropwise. The reaction was carried out at room tempeature for overnight. Saturated $Na_2S_2O_3$ solution (5 mL) was added to the mixture and stirred for 30 min. The mixture was extracted by EtOAc (10 mL) and washed with saturated NaCl

solution (3 × 10 mL). The organic layer was dried over anhydrous Na_2SO_4, filtered and concentrated under vacuum. The crude product was purified by silica gel column chromatography (CH_2Cl_2/EtOAc 1:2) to afford **2** (4.7 mg, 60%) and the C14-epimer (0.7 mg, 9%) as white solids.
Physical state: white solid;
Melting point: 223–225 °C;
Rf = 0.3 (1:3 CH_2Cl_2: EtOAc);
[α]D = -7.6 (c = 0.14, $CHCl_3$);
HRMS (ESI-TOF): calc'd for $C_{23}H_{35}O_5^+$ [M+H$^+$] 391.2479, found 391.2483;
^{1}H NMR (600 MHz, $CDCl_3$) δ 5.89 (s, 1H), 4.96 (dd, *J* = 17.9, 2.0 Hz, 1H), 4.80 (dd, *J* = 18.1, 1.8 Hz, 1H), 4.15 (s, 1H), 3.84 (t, *J* = 10.1 Hz, 1H), 2.87 (dd, *J* = 8.9, 5.8 Hz, 1H), 2.25 – 2.13 (m, 3H), 1.95 – 1.85 (m, 3H), 1.83 – 1.79 (m, 1H), 1.78 – 1.67 (m, 6H), 1.61 (td, *J* = 12.0, 3.8 Hz, 1H), 1.52 – 1.48 (m, 2H), 1.37 (dd, *J* = 14.4, 3.2 Hz, 1H), 1.32 – 1.26 (m, 3H), 1.18 (s, 1H), 1.11 – 1.09 (m, 1H), 1.08 (s, 3H), 0.91 (s, 3H);
^{13}C NMR (151 MHz, $CDCl_3$) δ 174.4, 173.5, 118.2, 84.9, 73.5, 68.3, 67.1, 50.6, 50.0, 49.7, 41.9, 41.0, 37.7, 36.8, 33.9, 33.7, 32.7, 29.0, 26.9, 26.8, 24.1, 21.7, 17.2.

i) Synthesis of sarmentogenin diacetate

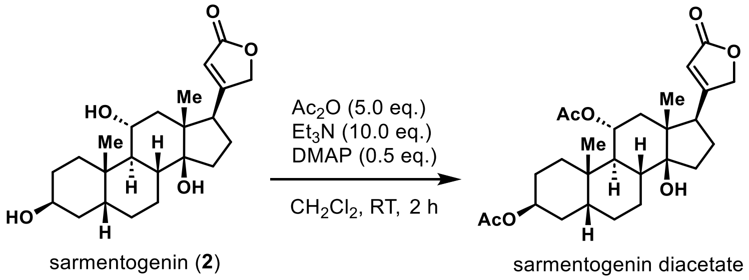

To a 5 mL tube was added compound **2** (2 mg, 0.005 mmol), DMAP (0.6 mg, 0.0025 mmol) and CH_2Cl_2 (1 mL). Then AC_2O (2 μL, 0.025 mmol) and Et_3N (7 μL, 0.05 mmol) were added sequentially. The resulting mixture was stirred at room temperature for 2 h. TLC showed the substrate was consumed completely. The solvent was removed under vacuum and the crude product was purified by silica gel column chromatography (CH_2Cl_2/EtOAc 2:1) to afford sarmentogenin diacetate (2.5 mg, 90%) as a white solid.
Physical state: white solid;
Melting point: 165–167 °C;
Rf = 0.5 (1:1 CH_2Cl_2: EtOAc);
[α]D = +12.7 °, (c = 0.25, $CHCl_3$);
HRMS (ESI-TOF): calc'd for $C_{27}H_{39}O_7^+$ [M+H$^+$] 475.2690, found 475.2694;
^{1}H NMR (600 MHz, $CDCl_3$) δ 5.88 (s, 1H), 5.12 – 5.08 (m, 1H), 4.97 (td, *J* = 10.6, 4.8 Hz, 1H), 4.92 (dd, *J* = 18.0, 1.8 Hz, 1H), 4.78 (dd, *J* = 18.0, 1.8 Hz, 1H), 2.78 (dd, *J* = 8.7, 5.9 Hz, 1H), 2.19 (dt, *J* = 12.4, 7.5 Hz, 2H), 2.05 (s, 3H), 2.03 – 1.98 (m, 1H), 1.95 (s, 3H), 1.93 – 1.62 (m, 10H), 1.53 – 1.36 (m, 4H), 1.34 – 1.27 (m, 2H), 1.22 (brs, 1H), 1.05 (s, 3H), 0.95 (s, 3H);
^{13}C NMR (151 MHz, $CDCl_3$) δ 174.3, 173.2, 170.9, 170.1, 118.3, 84.5, 73.4, 70.4, 70.3, 50.3, 49.5, 44.8, 40.9, 39.2, 38.4, 36.4, 33.5, 33.4, 31.0, 26.9, 26.6, 25.6, 23.9, 21.8, 21.7, 21.6, 16.6.
The NMR data was consistent with the reported literature.[2]

j) Synthesis of (2*R*,3*R*,4*R*,5*S*,6*S*)-2-(((3*S*,5*R*,8*R*,9*S*,10*S*,13*R*,17*S*)-10,13-dimethyl-11-oxo-17-(5-oxo-2,5-dihydrofuran-3-yl)-2,3,4,5,6,7,8,9,10,11,12,13,16,17-tetradecahydro-1*H*-cyclopenta[*a*]phenanthren-3-yl)oxy)-6-methyltetrahydro-2*H*-pyran-3,4,5-triyl tribenzoate (15)

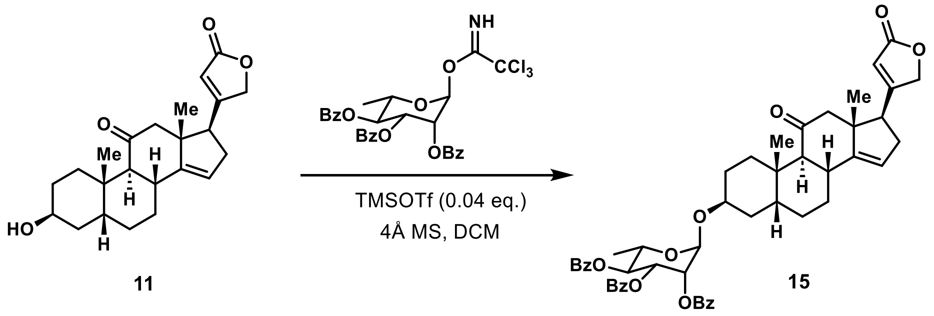

To a 10 mL dry flask was added compound **11** (10 mg, 0.027 mmol), 4 Å molecular sieves (10 mg) and dry CH_2Cl_2 (0.5 mL) under Ar. Then a solution of glycoside (33.5 mg, 0.054 mmol) in dry CH_2Cl_2 (0.5 mL) was added to the above solution by using a syringe and the mixture was stirred for 10 min at 0 °C Then TMSOTf (0.24 mg, 0.0011 mmol, dispersed in CH_2Cl_2) was added dropwise and the reaction mixture was stirred at the same temperature for 2 h. TLC analysis showed the material was consumed completely. Saturated $NaHCO_3$ solution (2 mL) was added to quench the reaction. Then, the mixture was extracted by EtOAc (3 × 10 mL) and washed with saturated NaCl solution (3 × 5 mL). The organic layer was dried over anhydrous Na_2SO_4, filtered and concentrated under vacuum. The crude product was purified by silica gel column chromatography (petroleum ether/EtOAc 1:1) to afford compound **15** (17.7 mg, 90%) as a white solid.

Physical state: white solid;

Melting point: 138–140 °C;

R_f = 0.1 (2:1 petroleum ether: EtOAc);

[α]D = +59.2, (c = 0.36, $CHCl_3$);

HRMS (ESI-TOF): calc'd for $C_{50}H_{56}O_{11}N^+$ [M+NH_4^+] 846.3848, found 846.3846;

^{1}H NMR (600 MHz, $CDCl_3$) δ 8.12 – 8.08 (m, 2H), 8.01 – 7.96 (m, 2H), 7.85 – 7.80 (m, 2H), 7.64 – 7.58 (m, 1H), 7.55 – 7.46 (m, 3H), 7.44 – 7.36 (m, 3H), 7.28 – 7.22 (m, 2H), 5.93 (d, *J* = 1.6 Hz, 0H), 5.84 (dd, *J* = 10.2, 3.5 Hz, 1H), 5.67 (t, *J* = 10.0 Hz, 1H), 5.62 (dd, *J* = 3.4, 1.8 Hz, 1H), 5.52 (m, 1H), 5.09 (d, *J* = 1.8 Hz, 1H), 4.78 (dd, *J* = 17.4, 1.8 Hz, 1H), 4.75 – 4.68 (m, 1H), 4.23 (dq, *J* = 9.8, 6.3 Hz, 1H), 4.03 (t, *J* = 2.7 Hz, 1H), 3.05 – 2.99 (m, 1H), 2.66 – 2.48 (m, 5H), 2.41 (d, *J* = 12.0 Hz, 1H), 2.16 (dt, *J* = 14.1, 3.4 Hz, 1H), 2.03 (tt, *J* = 14.0, 4.1 Hz, 1H), 1.90 (dtd, *J* = 13.3, 4.5, 4.0, 2.1 Hz, 1H), 1.81 – 1.57 (m, 6H), 1.46 (tt, *J* = 14.4, 3.3 Hz, 1H), 1.40 – 1.33 (m, 4H), 1.33 (s, 3H), 0.84 (s, 3H);

^{13}C NMR (151 MHz, $CDCl_3$) δ 208.9, 173.6, 169.0, 166.0, 165.9, 165.7, 150.6, 133.6, 133.5, 133.2, 130.0, 129.9, 129.8, 129.4, 129.4, 128.7, 128.6, 128.4, 119.6, 117.3, 96.3, 73.5, 73.3, 72.0, 71.7, 70.3, 67.1, 57.4, 52.8, 51.9, 50.5, 37.5, 35.5, 34.9, 34.2, 30.1, 30.0, 27.1, 25.8, 24.7, 23.6, 20.0, 17.8.

k) Synthesis of (2*R*,3*R*,4*R*,5*S*,6*S*)-2-(((3*S*,5*R*,8*R*,9*S*,10*S*,13*R*,14*S*,17*R*)-14-hydroxy-10,13-dimethyl-11-oxo-17-(5-oxo-2,5-dihydrofuran-3-yl)hexadecahydro-1*H*-cyclopenta[*a*]phenanthren-3-yl)oxy)-6-methyltetrahydro-2*H*-pyran-3,4,5-triyl tribenzoate (16)

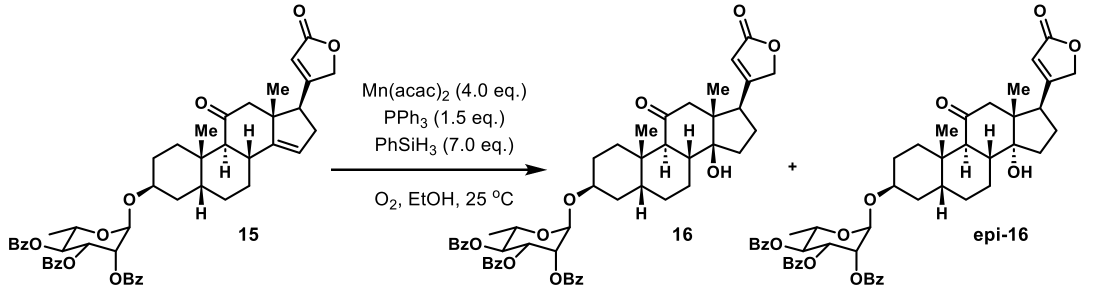

To a 10 mL tube was added compound **15** (24 mg, 0.03 mmol), $Mn(acac)_2$ (30 mg, 0.12 mmol) and PPh_3 (12 mg, 0.04 mmol). Then anhydrous ethanol (2.5 mL) was added under O_2 atmosphere. After being stirred for 5 min, $PhSiH_3$ (30 µL, 0.20 mmol) was added dropwise. The reaction was carried out at room tempeature for 12 h. Saturated $Na_2S_2O_3$ solution (10 mL) was added to the mixture and stirred for 30 min. The mixture was extracted by EtOAc (10 mL) and washed with saturated NaCl solution (3 × 10 mL). The organic layer was dried over anhydrous Na_2SO_4, filtered and concentrated under vacuum. The crude product was purified by silica gel column chromatography (CH_2Cl_2/EtOAc 1:2) to afford compound **16** (13.0 mg, 53%) and *epi*-**16** (6.6 mg, 27%) as white solids.

Compound 16

Physical state: white solid;

Melting point: 126–128 °C;

R_f = 0.75 (20:1 CH_2Cl_2: MeOH);

[α]D = +48.0 (c = 0.10, $CHCl_3$);

HRMS (ESI-TOF): calc'd for $C_{50}H_{58}O_{12}N^+$ [$M+NH_4^+$] 864.3953, found 864.3946;

^{1}H NMR (600 MHz, $CDCl_3$) δ 8.12 – 8.07 (m, 1H), 8.01 – 7.96 (m, 1H), 7.84 – 7.80 (m, 1H), 7.64 – 7.58 (m, 1H), 7.51 (dt, *J* = 15.5, 7.6 Hz, 2H), 7.42 (dd, *J* = 14.1, 6.7 Hz, 1H), 7.29 – 7.23 (m, 1H), 5.91 (d, *J* = 2.2 Hz, 0H), 5.84 (dd, *J* = 10.1, 3.4 Hz, 1H), 5.67 (t, *J* = 10.0 Hz, 1H), 5.62 (dd, *J* = 3.5, 1.8 Hz, 0H), 5.09 (d, *J* = 1.8 Hz, 0H), 4.87 – 4.75 (m, 1H), 4.23 (dq, *J* = 9.6, 6.2 Hz, 1H), 4.07 (t, *J* = 2.9 Hz, 1H), 2.58 (dd, *J* = 9.3, 6.5 Hz, 1H), 2.40 (dd, *J* = 25.5, 12.7 Hz, 1H), 2.26 – 2.18 (m, 1H), 2.10 (dt, *J* = 13.7, 3.1 Hz, 1H), 2.06 – 1.91 (m, 2H), 1.88 – 1.77 (m, 2H), 1.69 (tdd, *J* = 13.6, 5.8, 2.6 Hz, 1H), 1.65 – 1.60 (m, 2H), 1.43 – 1.36 (m, 1H), 1.34 (d, *J* = 6.2 Hz, 2H), 1.19 (s, 1H), 0.89 (s, 1H);

^{13}C NMR (151 MHz, $CDCl_3$) δ 211.7, 173.9, 170.7, 166.0, 166.0, 165.8, 133.7, 133.5, 133.3, 130.0, 129.9, 129.8, 129.6, 129.4, 129.3, 128.8, 128.6, 128.4, 118.1, 96.1, 83.8, 73.4, 73.2, 72.0, 71.7, 70.3, 67.1, 54.4, 51.6, 49.2, 47.7, 43.1, 37.1, 36.8, 34.5, 30.4, 30.4, 27.0, 26.6, 26.5, 24.1, 21.7, 18.7, 17.8.

Compound *epi*-16

Physical state: white solid;

Melting point: 117–119 °C;

R_f = 0.65 (20:1 CH_2Cl_2: MeOH);

[α]D = +87.1 (c = 0.20, $CHCl_3$);

HRMS (ESI-TOF): calc'd for $C_{50}H_{58}O_{12}N^+$ [$M+NH_4^+$] 864.3947, found 864.3953;

^{1}H NMR (400 MHz, $CDCl_3$) δ 8.13 – 8.06 (m, 2H), 8.04 – 7.93 (m, 2H), 7.85 – 7.79 (m, 2H), 7.61

(t, J = 7.5 Hz, 1H), 7.55 – 7.46 (m, 3H), 7.45 – 7.35 (m, 3H), 7.26 (t, J = 8.0 Hz, 2H), 5.88 (t, J = 1.8 Hz, 1H), 5.84 (dd, J = 10.2, 3.4 Hz, 1H), 5.67 (t, J = 10.0 Hz, 1H), 5.62 (dd, J = 3.4, 1.7 Hz, 1H), 5.08 (d, J = 1.7 Hz, 1H), 4.76 (dd, J = 17.5, 1.8 Hz, 1H), 4.66 (dd, J = 17.7, 1.8 Hz, 1H), 4.23 (dq, J = 9.5, 6.1 Hz, 1H), 4.04 (s, 1H), 3.40 (t, J = 8.6 Hz, 1H), 3.02 (d, J = 12.1 Hz, 1H), 2.94 (d, J = 11.5 Hz, 1H), 2.49 (dd, J = 13.1, 3.9 Hz, 1H), 2.27 – 2.19 (m, 1H), 2.13 (td, J = 11.9, 3.9 Hz, 1H), 2.04 – 1.87 (m, 4H), 1.82 – 1.61 (m, 7H), 1.56 – 1.43 (m, 3H), 1.34 (d, J = 6.3 Hz, 4H), 1.25 (s, 3H), 0.69 (s, 3H);

^{13}C NMR (151 MHz, $CDCl_3$) δ 210.1, 173.7, 170.5, 166.0, 165.9, 165.8, 133.6, 133.5, 133.2, 130.0, 129.9, 129.8, 129.6, 129.4, 129.4, 128.7, 128.6, 128.4, 117.2, 96.3, 84.0, 73.6, 73.4, 72.0, 71.7, 70.4, 67.1, 51.9, 50.0, 45.3, 45.0, 40.5, 37.7, 34.9, 33.5, 30.4, 30.2, 27.1, 26.0, 25.2, 23.4, 21.6, 18.4, 17.8.

l) Synthesis of intermediate 17 and rhodexin A (1)

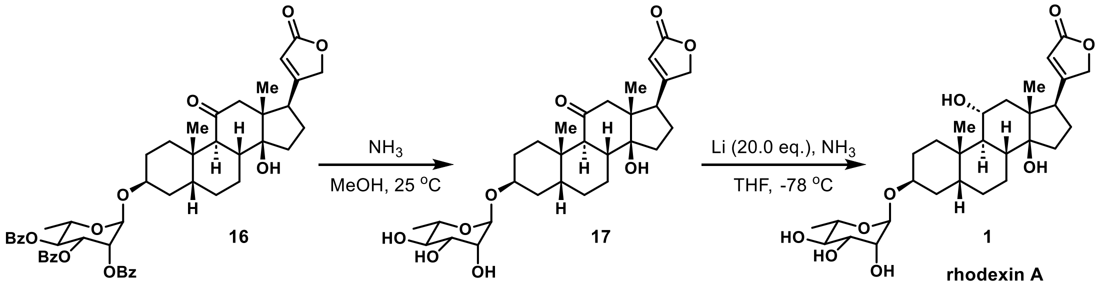

To a 10 mL dry tube was added compound **16** (3.5 mg, 0.004 mmol) and a solution of NH_3 in methanol (300 μL, 1 M). The mixture was stirred at room temperature for overnight. After completion, HCl (200μL, 2 M) was added to the flask and stirred for 2 h. Then the mixture was extracted by EtOAc (10 mL) and washed with saturated NaCl solution (3 × 10 mL). The organic layer was dried over anhydrous Na_2SO_4, filtered and concentrated under vacuum. The crude product was purified by silica gel column chromatography (CH_2Cl_2/MeOH 20:1) to afford compound **17** (2.3 mg, 95%).

Physical state: white solid;

Melting point: 108–110 °C;

$\boldsymbol{R_f}$ = 0.5 (5:1 CH_2Cl_2: MeOH);

[α]D = -10.0 (c = 0.14, $CHCl_3$);

HRMS (ESI-TOF): calc'd for $C_{29}H_{42}O_9Na^+$ [M+Na^+] 557.2721, found 557.2731;

^{1}H NMR (600 MHz, CD_3OD) δ 5.94 (q, J = 1.6 Hz, 1H), 4.95 (d, J = 1.8 Hz, 2H), 4.77 (d, J = 1.7 Hz, 1H), 3.94 (s, 1H), 3.69 (dd, J = 9.5, 3.3 Hz, 1H), 3.68 – 3.63 (m, 1H), 3.37 (t, J = 9.5 Hz, 1H), 2.70 (t, J = 8.1 Hz, 1H), 2.61 (d, J = 12.5 Hz, 1H), 2.46 (d, J = 13.0 Hz, 1H), 2.30 – 2.21 (m, 2H), 2.16 (td, J = 12.6, 3.7 Hz, 1H), 2.14 – 2.07 (m, 1H), 2.09 – 2.03 (m, 1H), 2.02 – 1.75 (m, 6H), 1.71 – 1.54 (m, 4H), 1.54 – 1.41 (m, 3H), 1.30 (d, J = 13.2 Hz, 3H), 1.24 (d, J = 6.2 Hz, 3H), 1.13 (s, 3H), 0.84 (s, 3H);

^{13}C NMR (151 MHz, CH_3OD) δ 213.8, 176.7, 175.4, 118.1, 99.9, 84.6, 75.2, 74.1, 73.3, 72.9, 72.5, 70.0, 55.3, 53.4, 50.7, 48.3, 44.1, 38.8, 37.2, 34.1, 31.4, 31.1, 27.9, 27.6, 27.6, 24.4, 22.9, 18.4, 18.0.

To a flask equipped with a dry-ice condenser containing ammonia at −78 °C was added lithium wire (2.0 mg, 0.28 mmol). After 10 minutes, compound **17** (2.0 mg, 0.0037 mmol) in THF (200 μL) was introduced to the blue ammonia solution at −78 °C. The reaction was allowed to stir for 3 min and then quenched with dropwise addition of THF/*t*-BuOH 10:1 (3 mL), followed by saturated aq. NH_4Cl (5 mL). After warming to room temperature, the aqueous layer was extracted with

$CHCl_3$/EtOH 4:1 (v/v). The organic layer was dried over anhydrous Na_2SO_4, filtered and concentrated under vacuum. The crude product was purified by silica gel column chromatography (CH_2Cl_2/MeOH 10:1) to afford rhodexin A (1.2 mg, 60%) as a white solid.
Physical state: white solid;
Melting point: 240–242 °C;
R_f = 0.2 (5:1 CH_2Cl_2: MeOH);
$[\alpha]_D$ = -21.5 (c = 0.1, MeOH);
HRMS (ESI-TOF): calc'd for $C_{29}H_{48}NO_9^+$ [$M+NH_4^+$] 554.3327, found 554.3324;
^{1}H NMR (400 MHz, Pyrdine-d5) δ 6.13 (s, 1H), 5.60 (brs, 1H), 5.46 (s, 1H), 5.43 (s, 1H), 5.31 (d, *J* = 17.9 Hz, 1H), 5.05 (d, *J* = 18.0 Hz, 1H), 4.92 – 4.56 (m, 2H), 4.33 (t, *J* = 6.4 Hz, 2H), 4.27 (s, 1H), 4.11 (brs, 1H), 3.07 – 2.92 (m, 2H), 2.36 – 2.06 (m, 6H), 2.05 – 1.72 (m, 11H) 1.66 (d, *J* = 5.5 Hz, 3H), 1.49 – 1.43 (m, 1H), 1.30 – 1.29 (m, 3H), 1.13 (s, 3H), 1.13 (s, 3H).
^{13}C NMR (101 MHz, Pyridine-d5) δ 175.9, 174.9, 118.1, 100.5, 84.6, 74.6, 74.1, 73.5, 73.4, 73.4, 70.5, 68.1, 51.7, 51.0, 50.7, 42.7, 41.7, 39.2, 37.4, 34.2, 34.0, 31.4, 28.5, 28.0, 27.7, 24.9, 22.6, 19.0, 18.0.

The NMR data and optical rotation were consistent with the reported literatures.[2,3]

3. Data comparison

a) Comparison of ^{1}H NMR data of sarmentogenin diacetate

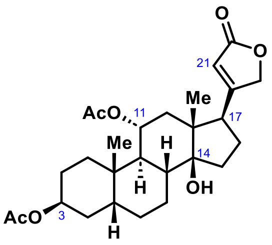

sarmentogenin diacetate

Lit[2]<br>δ ^{1}H NMR<br>500 MHz, $CDCl_3$	Synthetic<br>δ ^{1}H NMR<br>600 MHz, CD_3OD
5.88 (s, 1H)	5.88 (s, 1H)
5.13-5.07 (m, 1H)	5.12-5.08 (m, 1H)
4.97 (ddd, J = 11.0, 11.0, 5.0, 1H)	4.97 (td, *J* = 10.6, 4.8 Hz, 1H)
4.92 (d, J = 18.5 Hz, 1H)	4.92 (dd, *J* = 18.0, 1.8 Hz, 1H)
4.78 (d, J = 18.5 Hz, 1H)	4.78 (dd, *J* = 18.0, 1.8 Hz, 1H)
2.80-2.76 (m, 1H)	2.78 (dd, *J* = 8.7, 5.9 Hz, 1H)
2.05 (s, 3H)	2.05 (s, 3H)
1.95 (s, 3H)	1.95 (s, 3H)
1.05 (s, 3H)	1.05 (s, 3H)
0.95 (s, 3H)	0.95 (s, 3H)

b) Comparison of ^{13}C NMR data of sarmentogenin diacetate

**Lit**[2]<br>δ ^{13}C NMR<br>125 MHz, $CDCl_3$	**Synthetic**<br>δ ^{13}C NMR<br>151 MHz, $CDCl_3$
16.3	16.6
21.3	21.6
21.4	21.7
21.5	21.8
23.6	23.9
25.3	25.6
26.3	26.6
26.6	26.9
30.7	31.0
33.1	33.4
33.2	33.5
36.2	36.4
38.1	38.4
38.9	39.2
40.6	40.9
44.5	44.8
49.2	49.5
50.1	50.3
70.0	70.3
70.2	70.4
73.1	73.4
84.3	84.5
118.1	118.3
169.9	170.1
170.6	170.9
172.9	173.2
174.0	174.3

c) Comparison of ^{1}H NMR data of rhodexin A

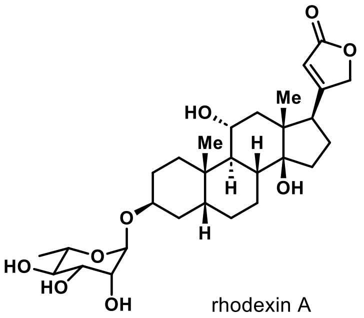

rhodexin A

**Lit[2]**<br>δ ^{1}H NMR<br>500 MHz, pyridine-d5	**Synthetic**<br>δ ^{1}H NMR<br>400 MHz, pyridine-d5
6.10 (s, 1H)	6.10 (s, 1H)
5.57 （brs, 1H)	5.57 (brs, 1H)
5.43 (s, 1H)	5.44 (s, 1H)
5.40 (s, 1H)	5.40 (s, 1H)
5.28 (d, J = 18.0 Hz, 1H)	5.28 (d, *J* = 17.9 Hz, 1H)
5.02 (d, J = 18.0 Hz, 1H)	5.02 (d, *J* = 18.1 Hz, 1H)
1.10 (s, 3H)	1.11 (s, 3H)
1.09 (s, 3H)	1.10 (s, 3H)

d) Comparison of ^{13}C NMR data of rhodexin A

**Lit[2]**<br>δ ^{13}C NMR<br>125 MHz, pyrdine-d_5	**Synthetic**<br>δ ^{13}C NMR<br>101 MHz, pyrdine-d_5
174.5	174.5
173.5	173.6
116.8	116.8
99.1	99.2
83.3	83.3
73.2	73.3
72.8	72.8
72.1	72.2
72.1	72.1
72.0	72.1
69.2	69.2
66.8	66.8
50.3	50.4
49.7	49.7
49.3	49.4
41.3	41.4
40.3	40.4
37.9	37.9
36.0	36.1
32.9	32.9
32.7	32.7
30.1	30.1
27.2	27.2
26.7	26.7
26.3	26.4
23.5	23.5
21.3	21.3
17.7	17.7
16.7	16.7

Comparison of specific rotation data of rhodexin A

**Lit[3]**<br>**$[\alpha]_D$**<br>(*c* 0.11, MeOH)	**Synthetic**<br>**$[\alpha]_D$**<br>(*c* 0.1, MeOH)
−17.0	−21.5

4. References

[1]. Song, F.; Zheng, M.; Wang, J.; Liu, H.; Lin, Z.; Liu, B.; Deng, Z.; Cong, H.; Zhou, Q.; Qu, X. Chemoenzymatic synthesis of C14-functionalized steroids. [J] *Nature Synthesis* **2023**, *2*, 729-739.

[2]. Jung, M. E.; Yoo, D. First Total Synthesis of Rhodexin A. [J] *Org Lett* **2011**, *13*, 3766-3766.

[3]. Masuda T.; Oyama Y.; Yamamoto N.; Umebayashi C.; Nakao H.; Toi Y.; Takeda Y.; Nakamoto K.; Kuninaga H.; Nishizato Y.; Nonaka A. Biosci. Biotechnol. Biochem. 2003, 67, 1401 – 1404

5. Copies of NMR spectra

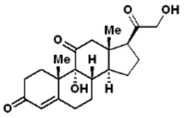

4'

^{1}H NMR, $CDCl_3$,

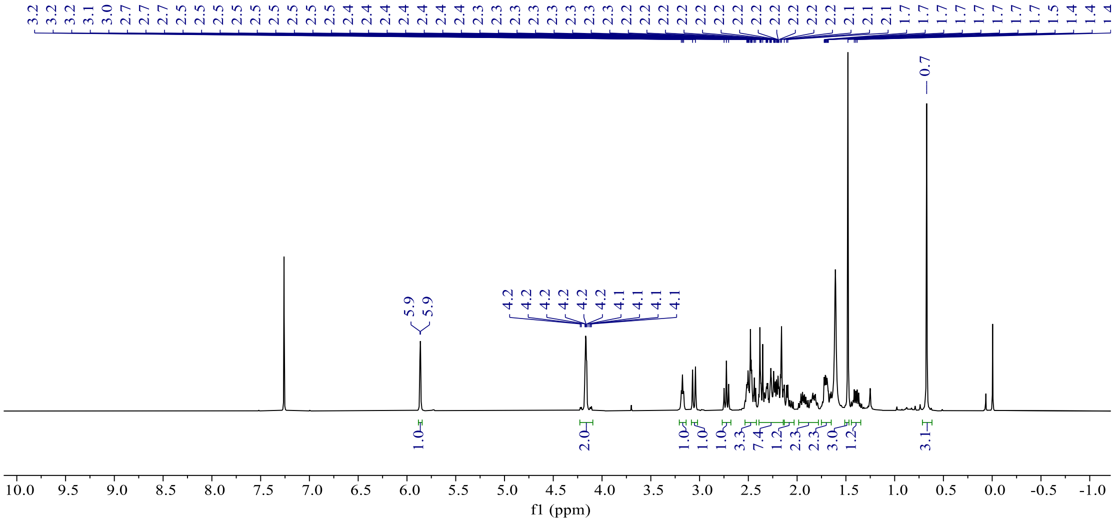

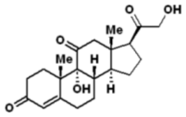

4'

^{13}C NMR, $CDCl_3$,

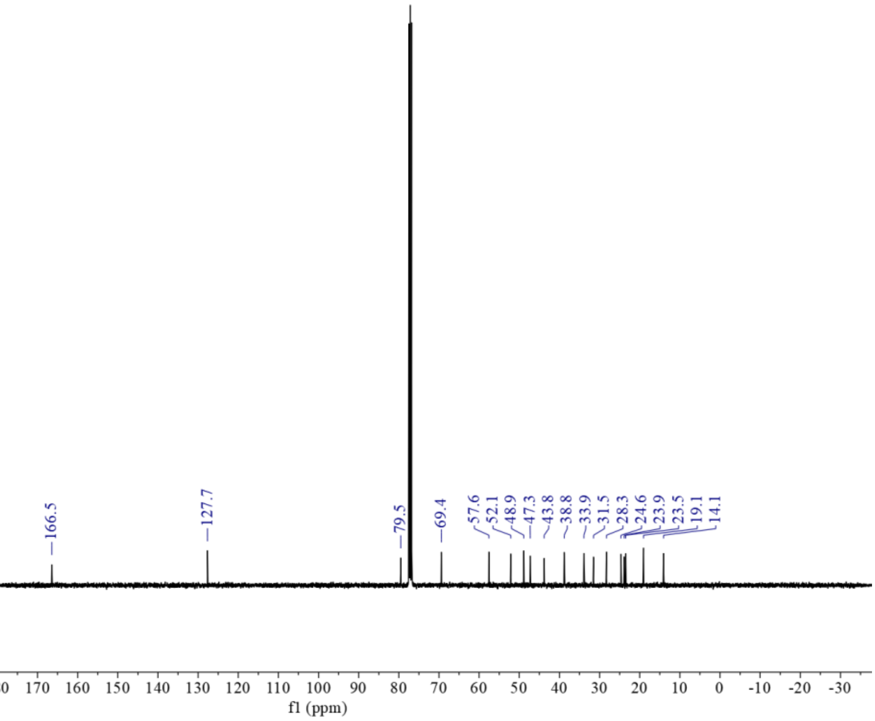

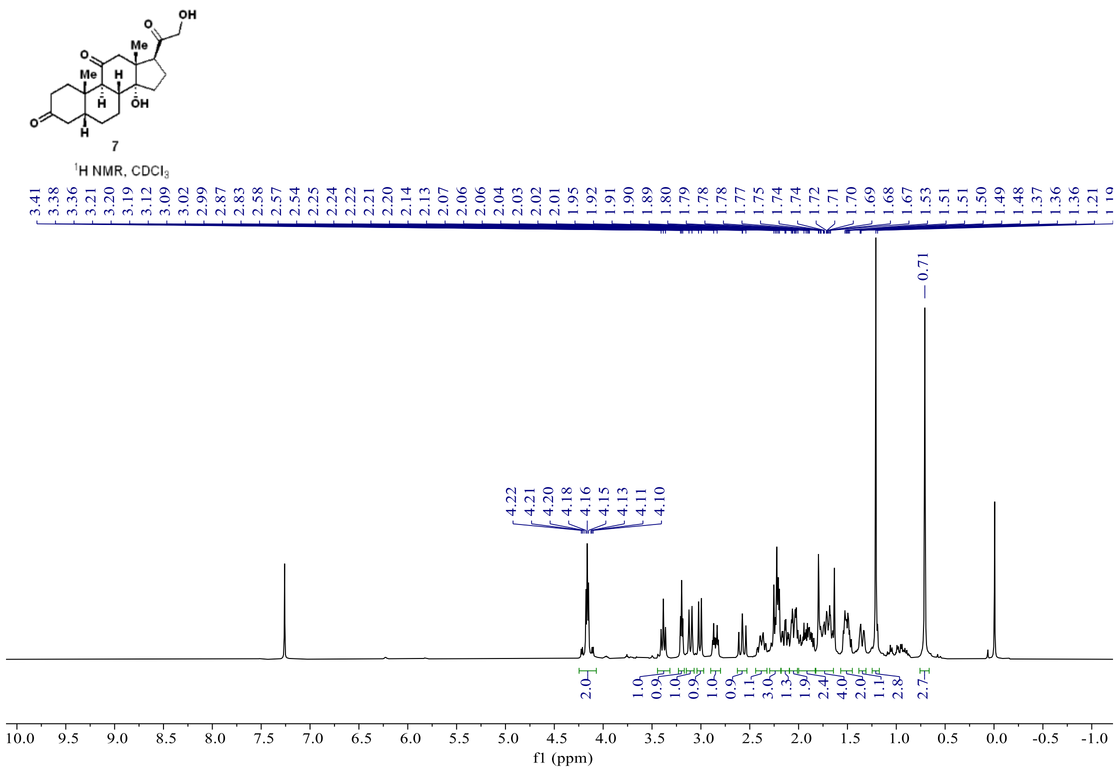
7
¹H NMR, CDCl3
f1 (ppm)

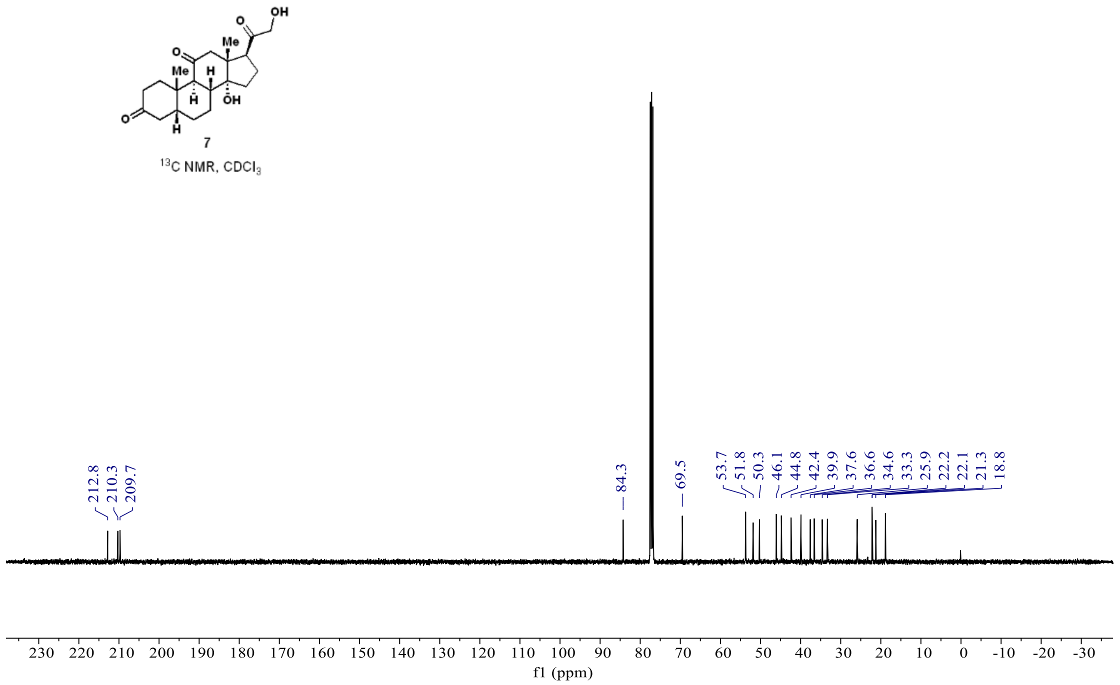
7
¹³C NMR, CDCl3
212.8
210.3
209.7
84.3
69.5
53.7
51.8
50.3
46.1
44.8
42.4
39.9
37.6
36.6
34.6
33.3
25.9
22.2
22.1
21.3
18.8
f1 (ppm)

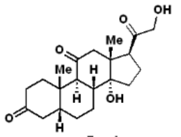

7-epi

^{1}H NMR, $CDCl_3$

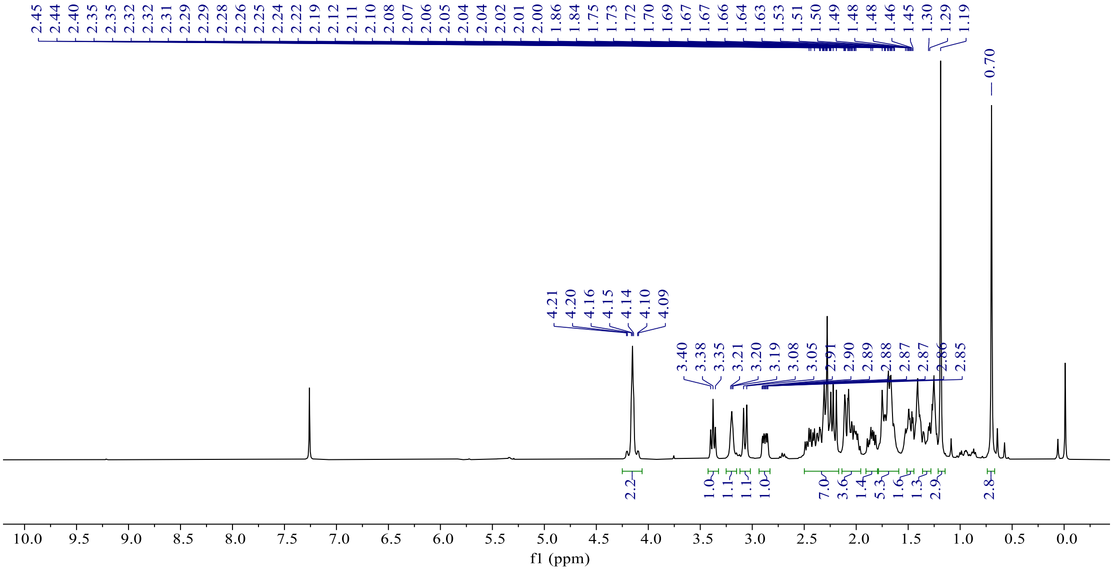

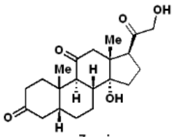

7-epi

^{13}C NMR, $CDCl_3$

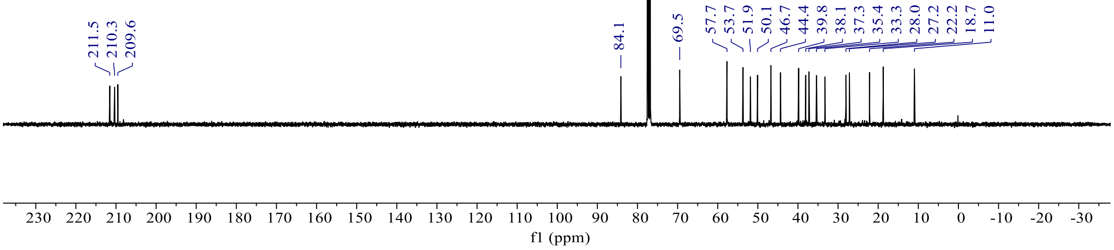

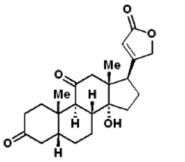

8

^1H NMR, $CDCl_3$

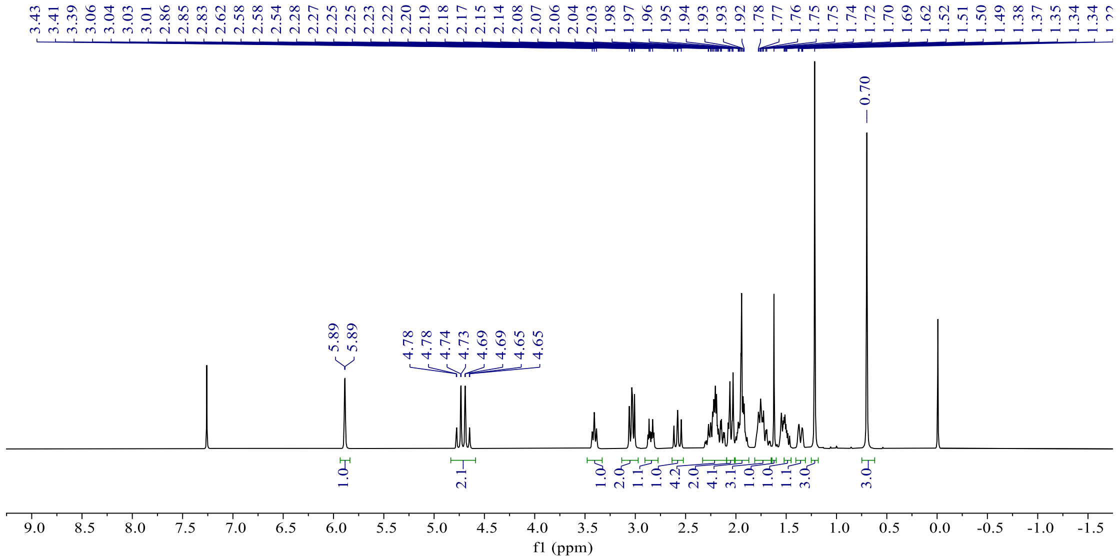

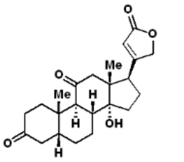

8

^{13}C NMR, $CDCl_3$

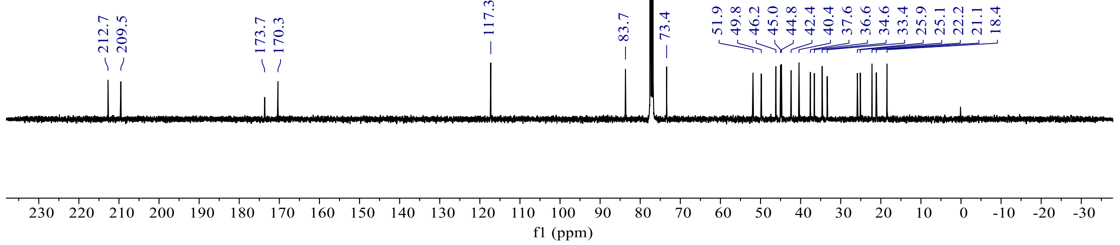

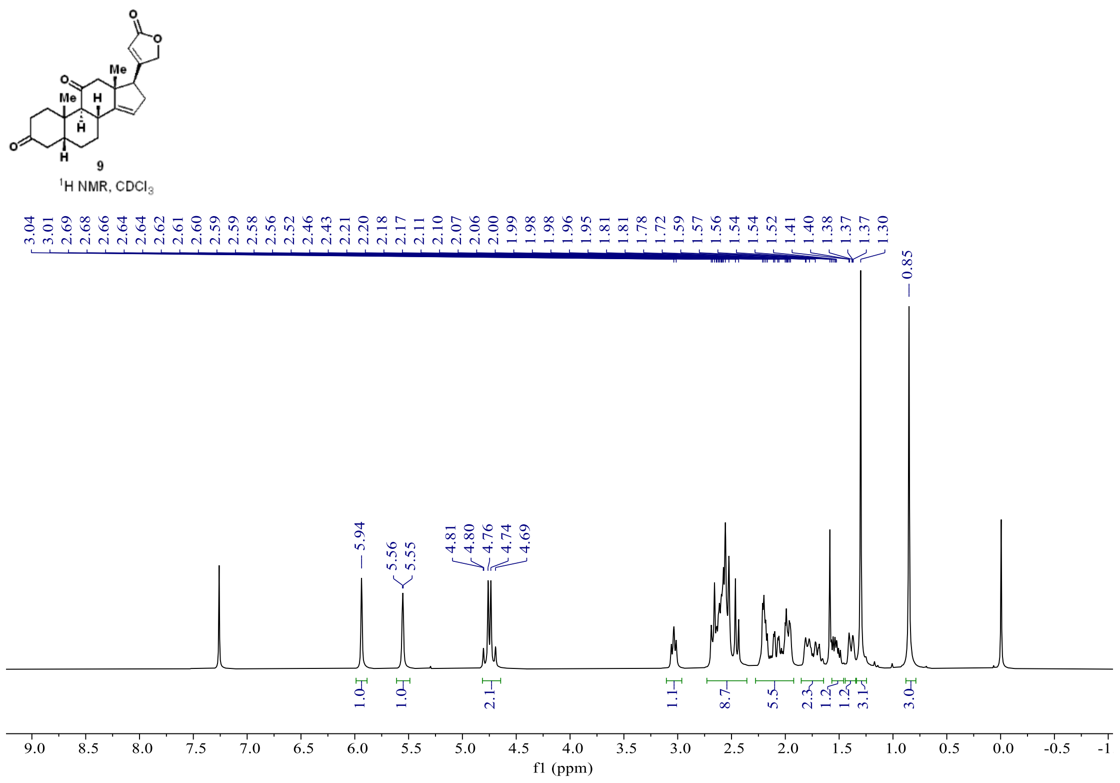

9
^{1}H NMR, CDCl$_3$
3.04 3.01 2.69 2.68 2.66 2.64 2.64 2.62 2.61 2.60 2.59 2.59 2.58 2.56 2.52 2.46 2.43 2.21 2.20 2.18 2.17 2.11 2.10 2.07 2.06 2.00 1.99 1.98 1.98 1.96 1.95 1.81 1.81 1.78 1.72 1.59 1.57 1.56 1.54 1.54 1.52 1.41 1.40 1.38 1.37 1.37 1.30
0.85
5.94
5.56 5.55
4.81 4.80 4.76 4.74 4.69
1.0 1.0 2.1 1.1 8.7 5.5 2.3 1.2 1.2 3.1 3.0
9.0 8.5 8.0 7.5 7.0 6.5 6.0 5.5 5.0 4.5 4.0 3.5 3.0 2.5 2.0 1.5 1.0 0.5 0.0 -0.5 -1
f1 (ppm)

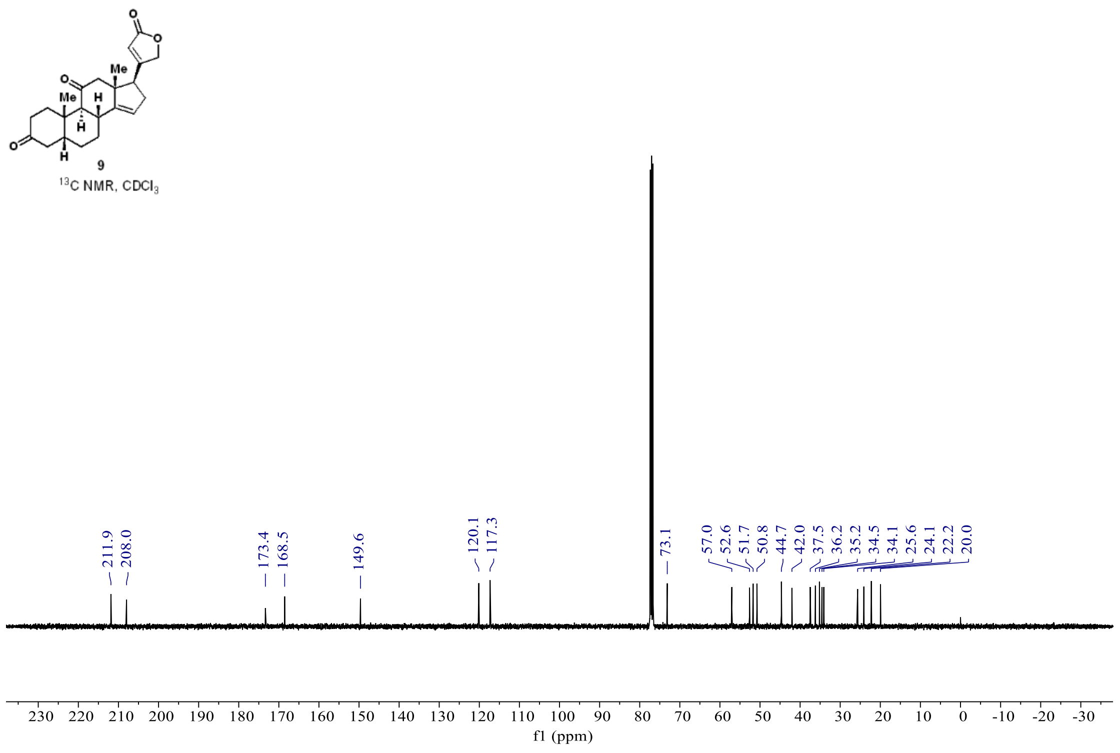

9
^{13}C NMR, CDCl$_3$
211.9 208.0
173.4 168.5
149.6
120.1 117.3
73.1
57.0 52.6 51.7 50.8 44.7 42.0 37.5 36.2 35.2 34.5 34.1 25.6 24.1 22.2 20.0
230 220 210 200 190 180 170 160 150 140 130 120 110 100 90 80 70 60 50 40 30 20 10 0 -10 -20 -30
f1 (ppm)

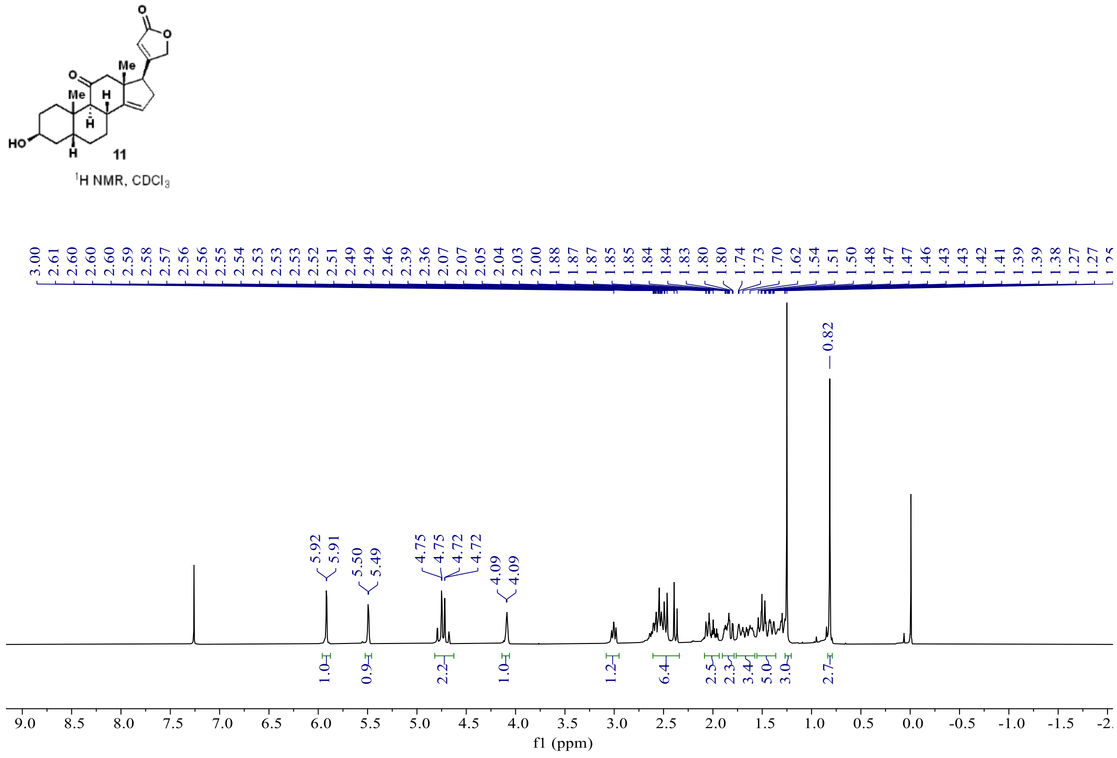

O
O
Me
O
Me
H
H
HO
H
11
^{1}H NMR, CDCl$_3$
3.00 2.61 2.60 2.60 2.60 2.59 2.58 2.57 2.56 2.55 2.54 2.53 2.53 2.53 2.52 2.51 2.49 2.49 2.46 2.39 2.36 2.07 2.07 2.05 2.04 2.03 2.00 1.88 1.87 1.87 1.85 1.85 1.84 1.84 1.83 1.80 1.80 1.74 1.73 1.70 1.62 1.54 1.51 1.50 1.48 1.47 1.47 1.46 1.43 1.43 1.42 1.41 1.39 1.38 1.27 1.27
0.82
5.92 5.91
5.50 5.49
4.75 4.75 4.72 4.72
4.09 4.09
1.0 0.9 2.2 1.0 1.2 6.4 2.5 2.3 3.4 5.0 3.0 2.7
9.0 8.5 8.0 7.5 7.0 6.5 6.0 5.5 5.0 4.5 4.0 3.5 3.0 2.5 2.0 1.5 1.0 0.5 0.0 -0.5 -1.0 -1.5
f1 (ppm)

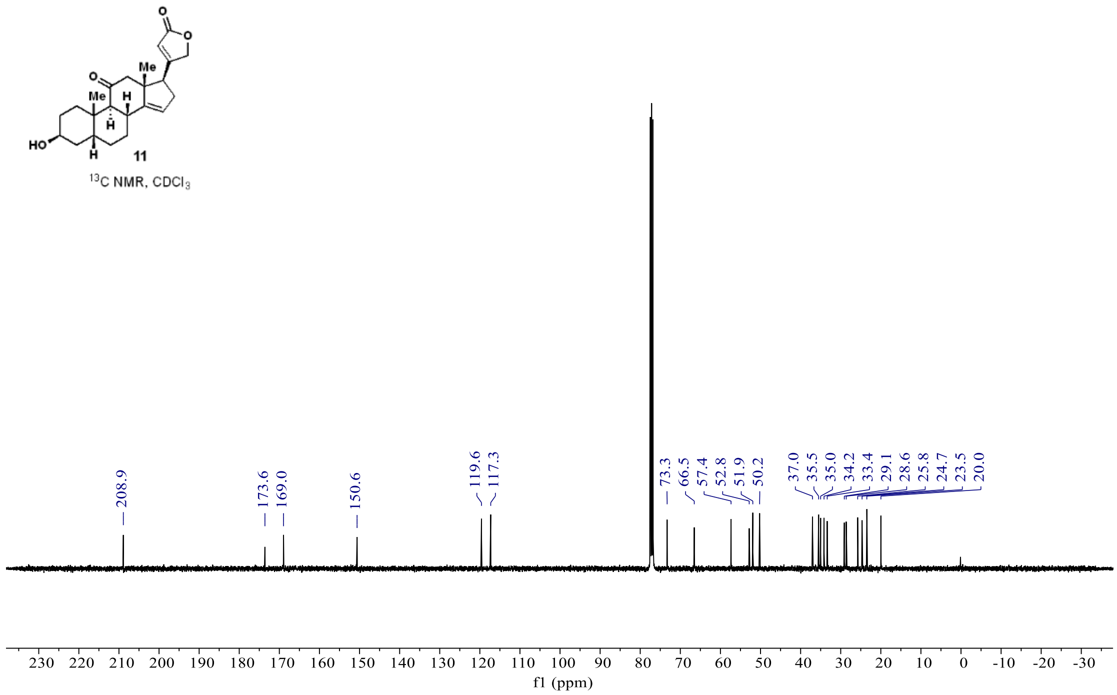

O
O
Me
O
Me
H
H
HO
H
11
^{13}C NMR, CDCl$_3$
208.9
173.6 169.0
150.6
119.6 117.3
73.3 66.5 57.4 52.8 51.9 50.2
37.0 35.5 35.0 34.2 33.4 29.1 28.6 25.8 24.7 23.5 20.0
230 220 210 200 190 180 170 160 150 140 130 120 110 100 90 80 70 60 50 40 30 20 10 0 -10 -20 -30
f1 (ppm)

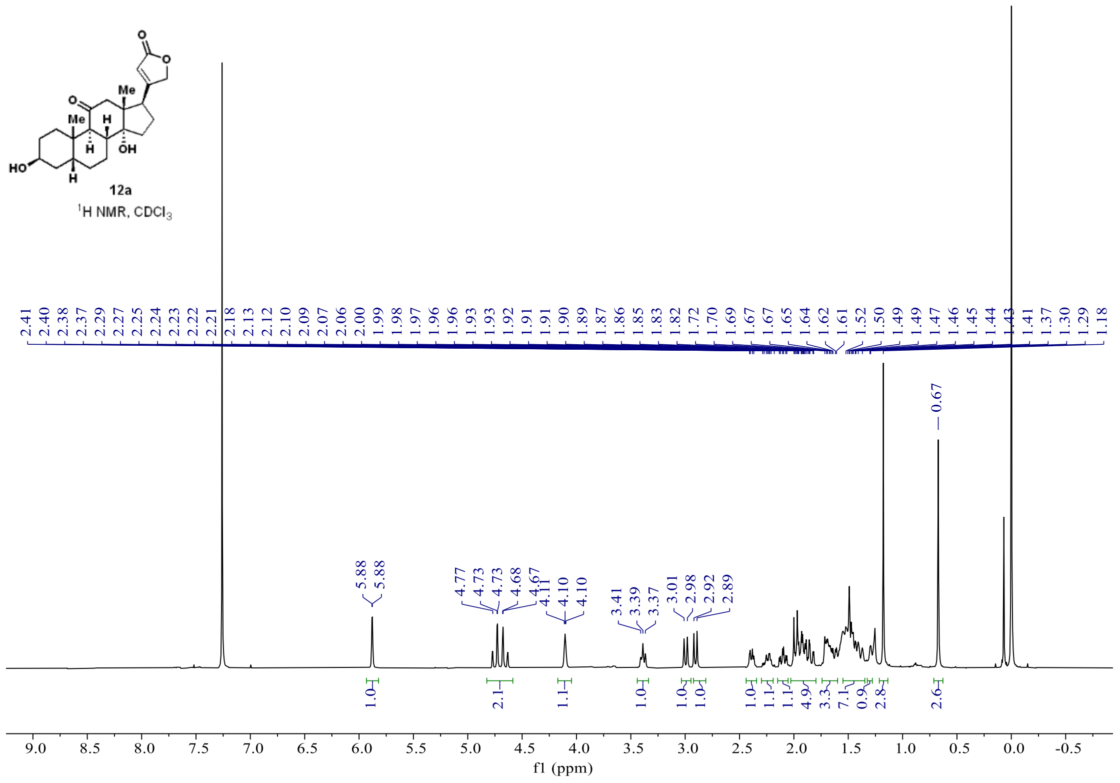
12a
1H NMR, CDCl3
f1 (ppm)

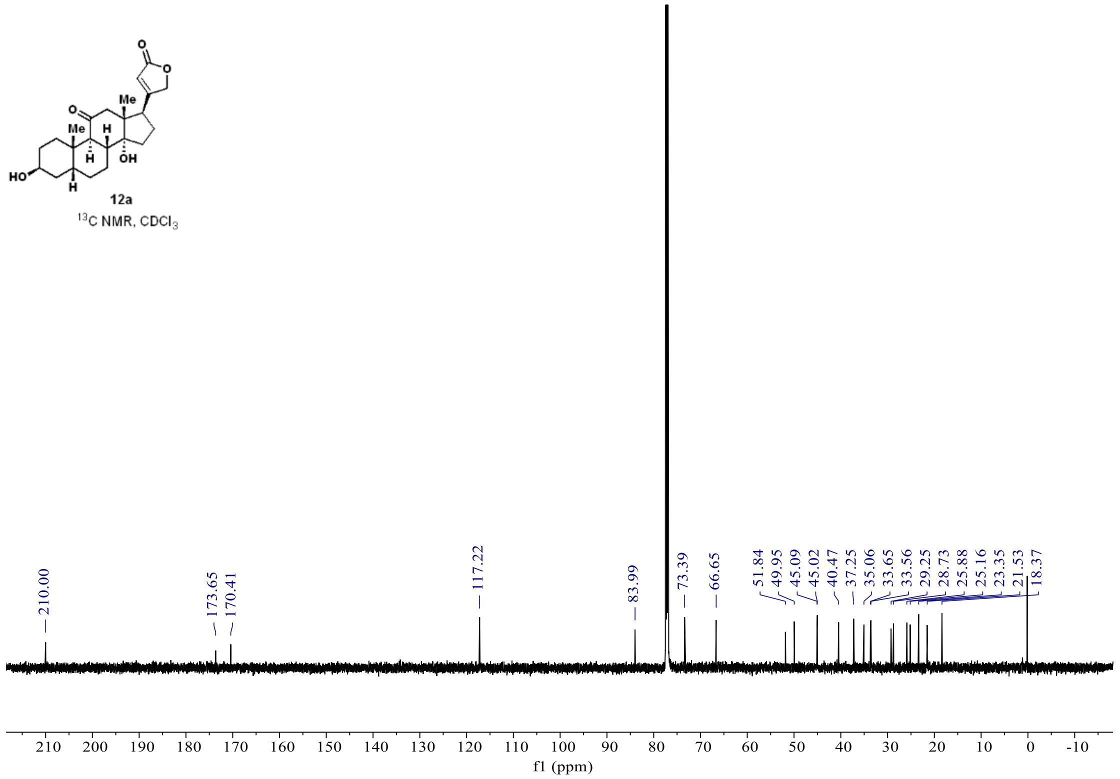
12a
13C NMR, CDCl3
210.00
173.65
170.41
117.22
83.99
73.39
66.65
51.84
49.95
45.09
45.02
40.47
37.25
35.06
33.65
33.56
29.25
28.73
25.88
25.16
23.35
21.53
18.37
f1 (ppm)

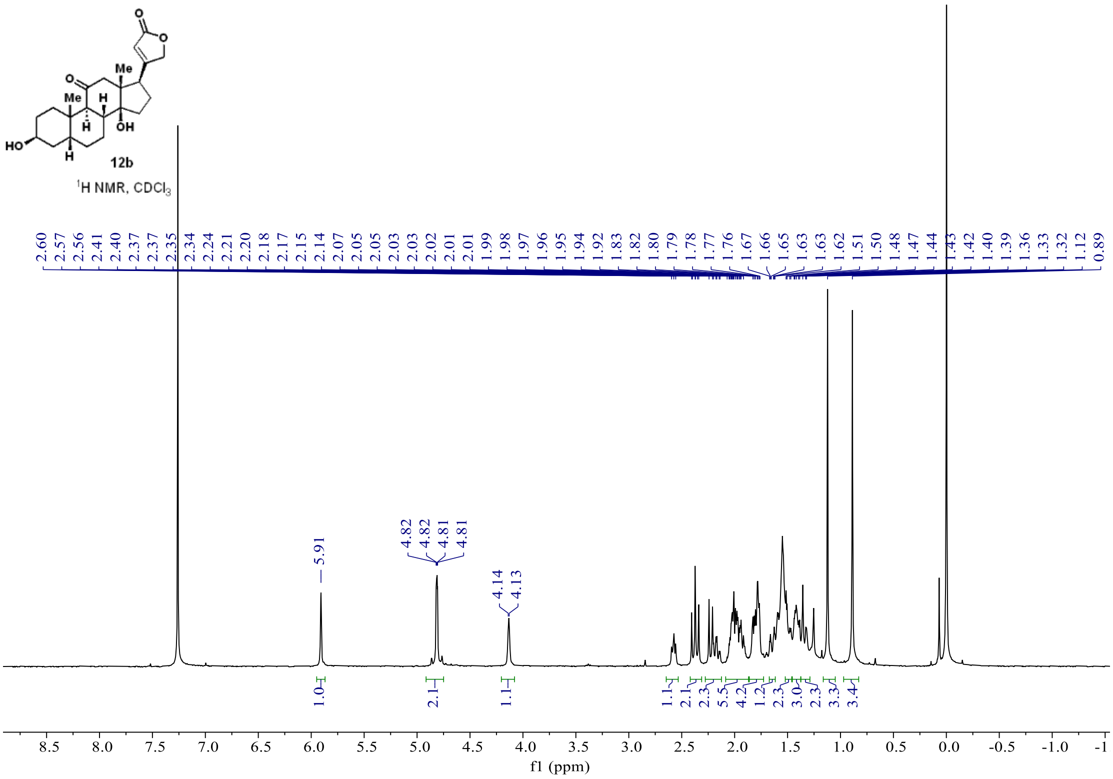
12b
^{1}H NMR, CDCl$_3$
2.60 2.57 2.56 2.41 2.40 2.37 2.37 2.35 2.34 2.24 2.21 2.20 2.18 2.17 2.15 2.14 2.07 2.05 2.05 2.03 2.03 2.02 2.01 2.01 1.99 1.98 1.97 1.96 1.95 1.94 1.92 1.83 1.82 1.80 1.79 1.78 1.77 1.76 1.67 1.66 1.65 1.63 1.63 1.62 1.51 1.50 1.48 1.47 1.44 1.43 1.42 1.40 1.39 1.36 1.33 1.32 1.12 0.89
5.91
4.82 4.82 4.81 4.81
4.14 4.13
1.0 2.1 1.1 1.1 2.1 2.3 5.5 4.2 1.2 2.3 3.0 2.3 3.3 3.4
8.5 8.0 7.5 7.0 6.5 6.0 5.5 5.0 4.5 4.0 3.5 3.0 2.5 2.0 1.5 1.0 0.5 0.0 -0.5 -1.0 -1
f1 (ppm)

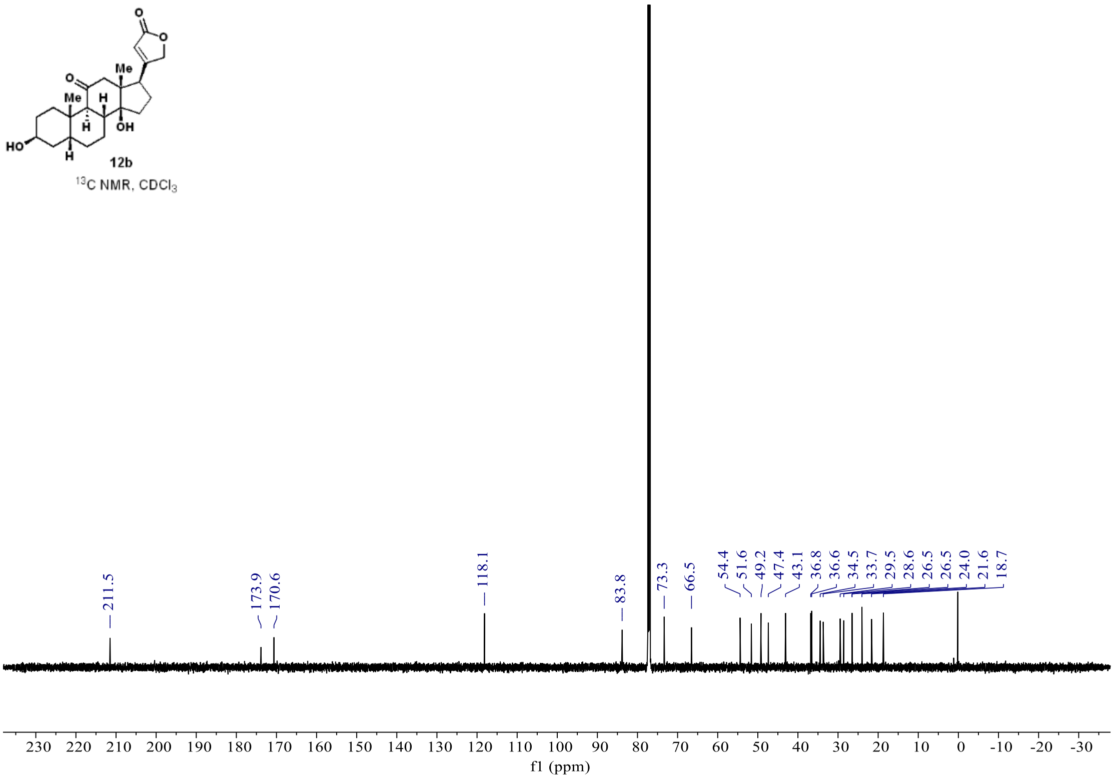
12b
^{13}C NMR, CDCl$_3$
211.5
173.9 170.6
118.1
83.8
73.3
66.5
54.4 51.6 49.2 47.4 43.1 36.8 36.6 34.5 33.7 29.5 28.6 26.5 26.5 24.0 21.6 18.7
230 220 210 200 190 180 170 160 150 140 130 120 110 100 90 80 70 60 50 40 30 20 10 0 -10 -20 -30
f1 (ppm)

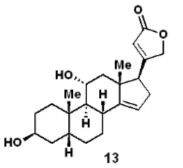

13

^{1}H NMR, $CDCl_3$

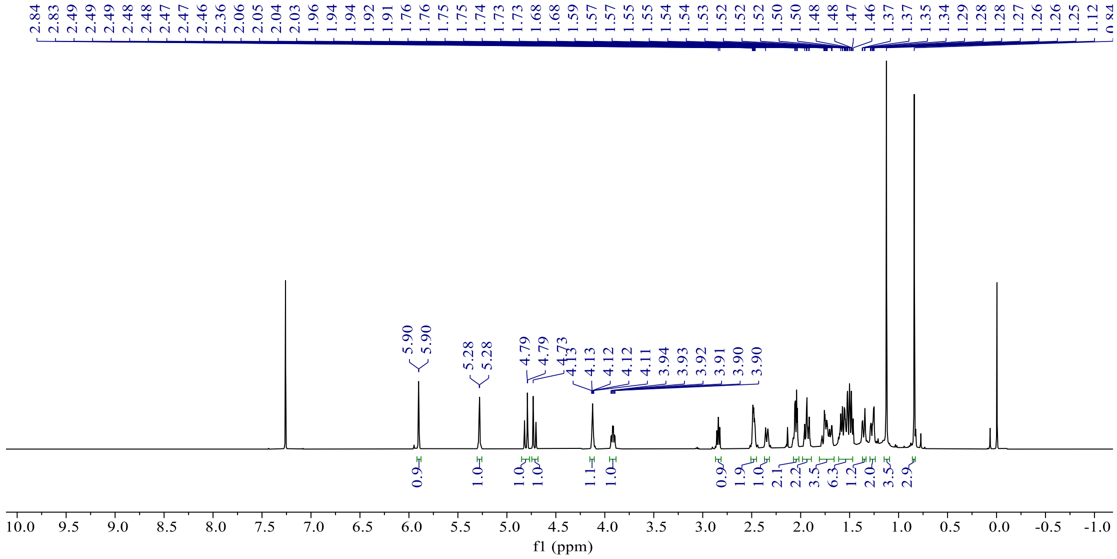

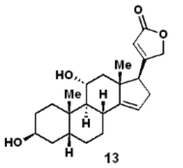

13

^{13}C NMR, $CDCl_3$

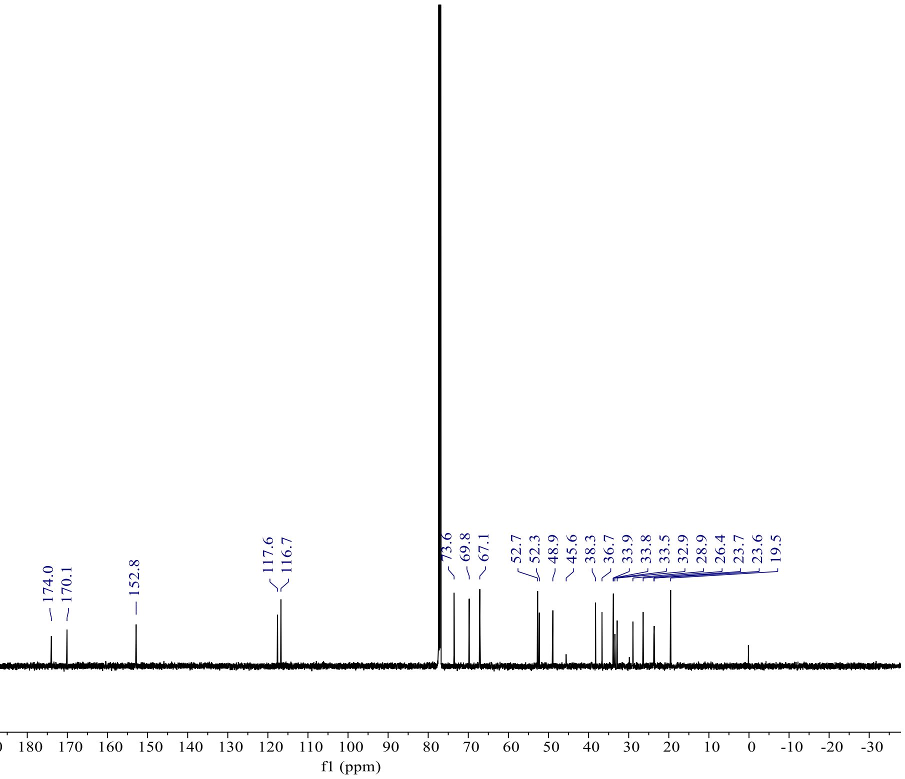

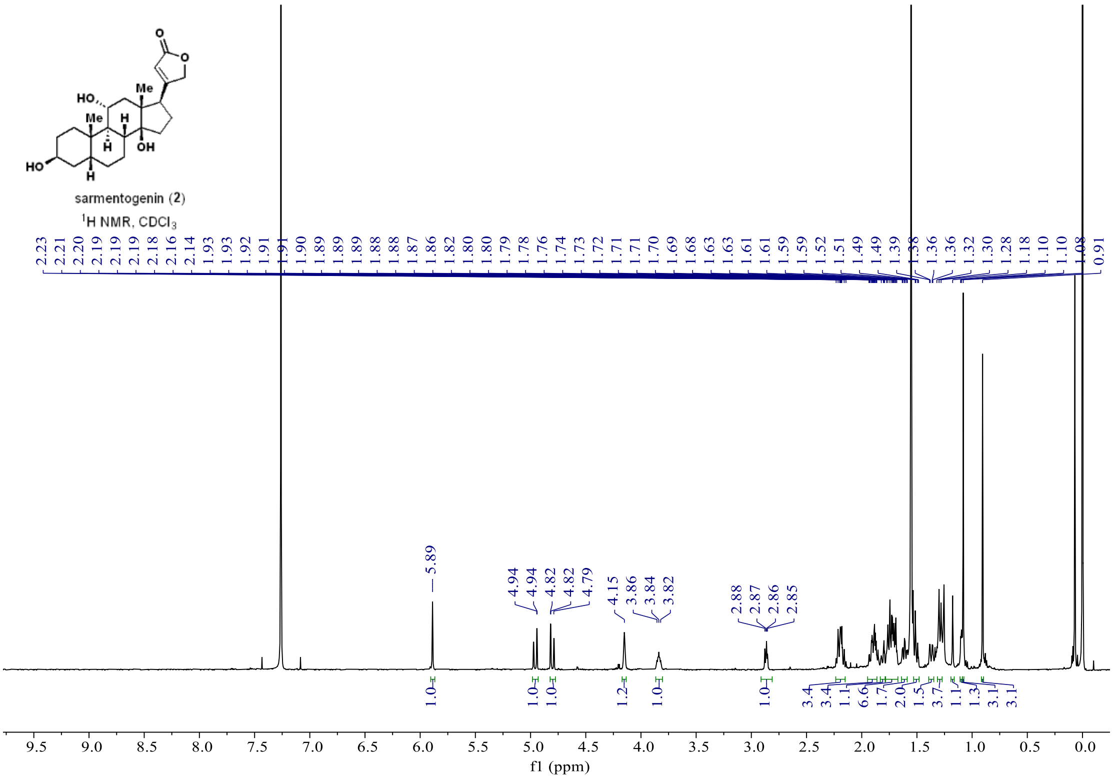
sarmentogenin (2)
¹H NMR, CDCl₃
f1 (ppm)

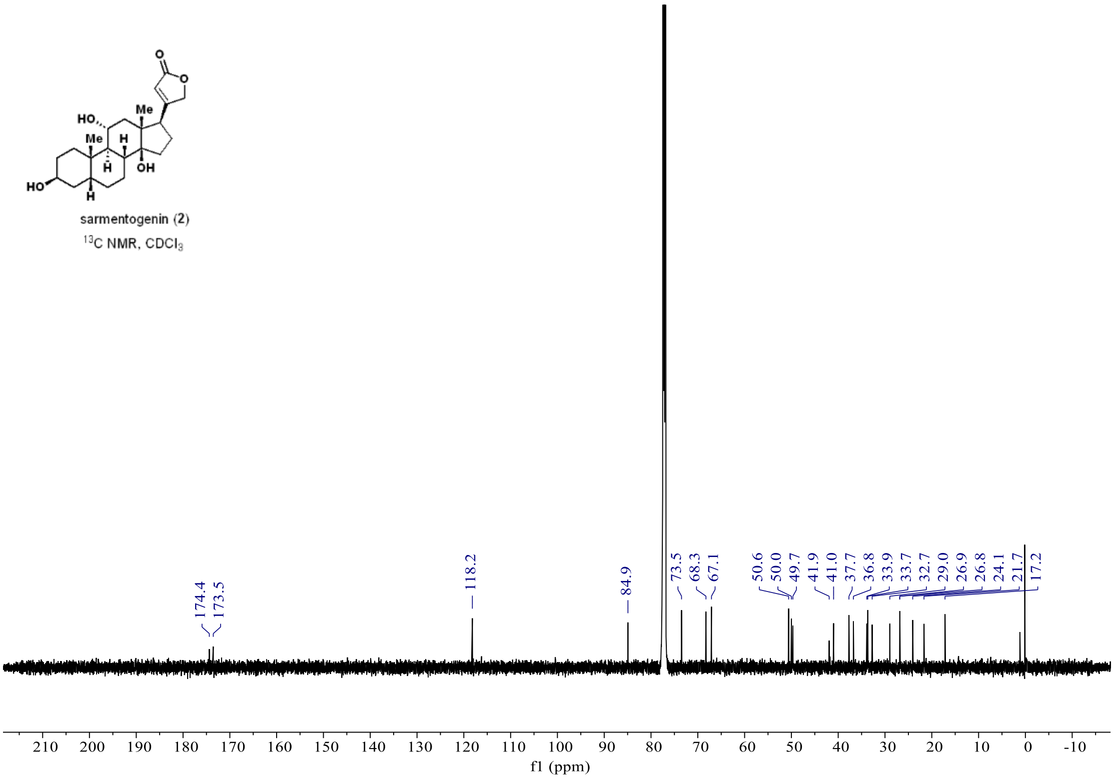
sarmentogenin (2)
¹³C NMR, CDCl₃
f1 (ppm)

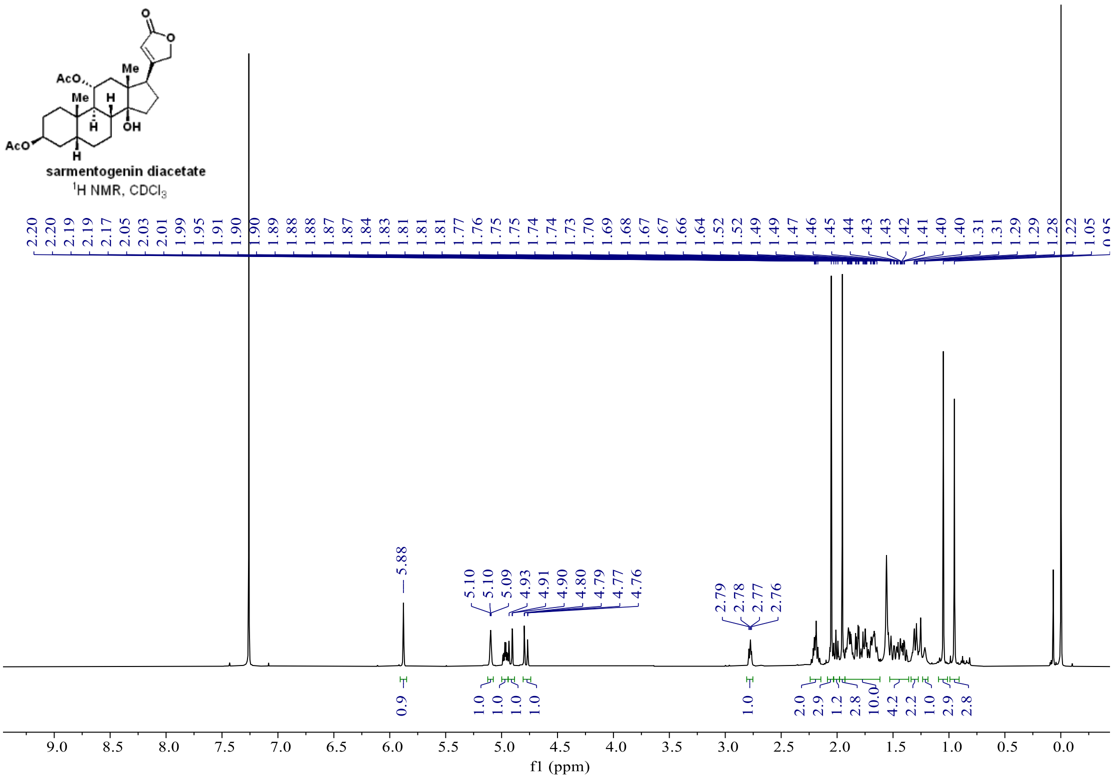
sarmentogenin diacetate
1H NMR, CDCl3
f1 (ppm)

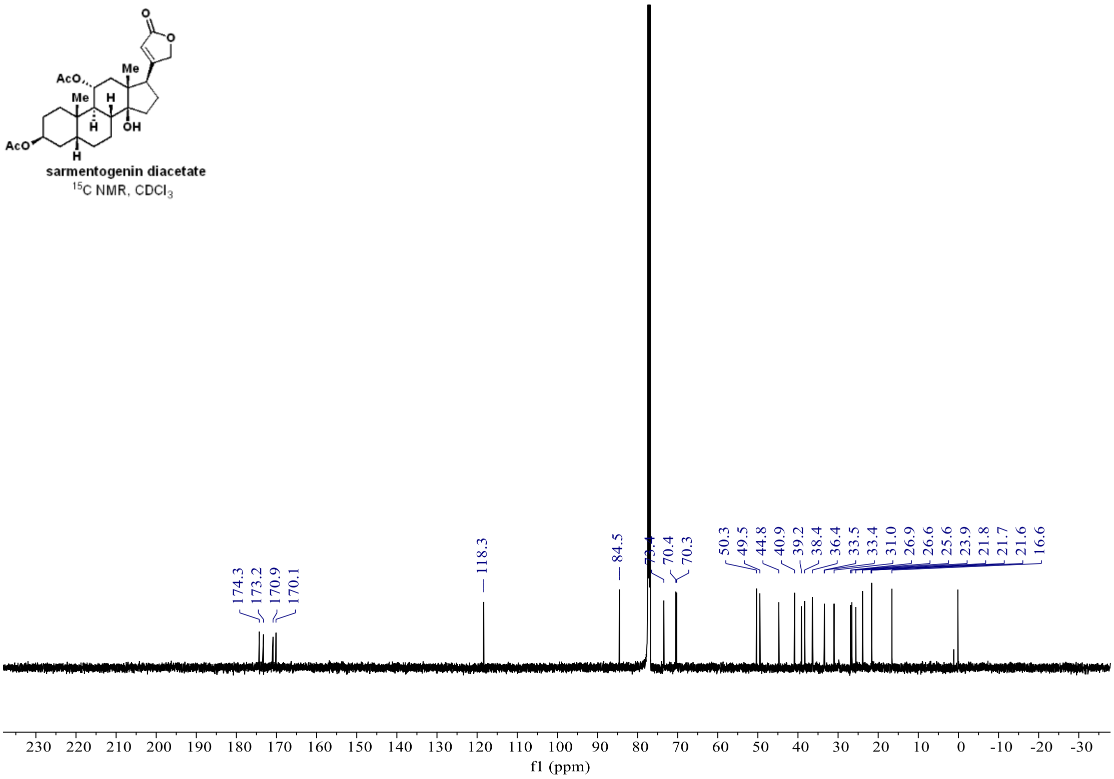
sarmentogenin diacetate
15C NMR, CDCl3
f1 (ppm)

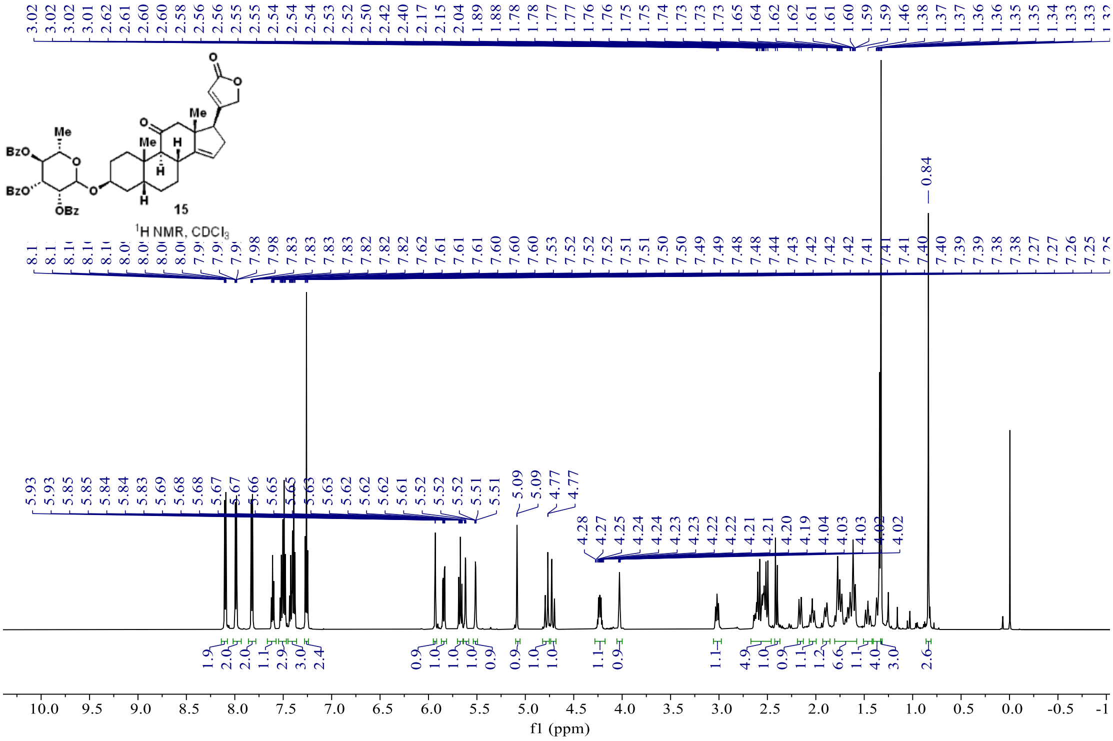
15
1H NMR, CDCl3
f1 (ppm)

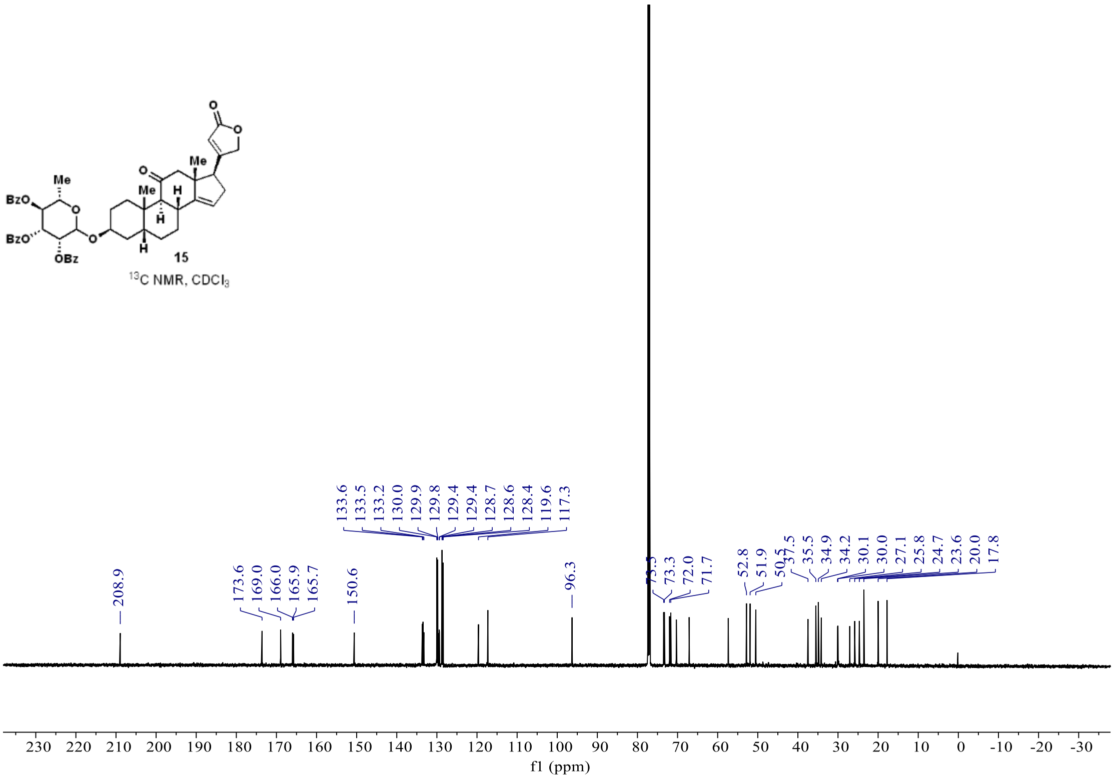
15
13C NMR, CDCl3
f1 (ppm)

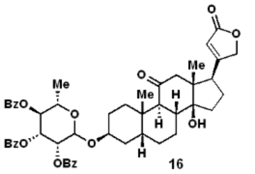

16

^{1}H NMR, $CDCl_3$

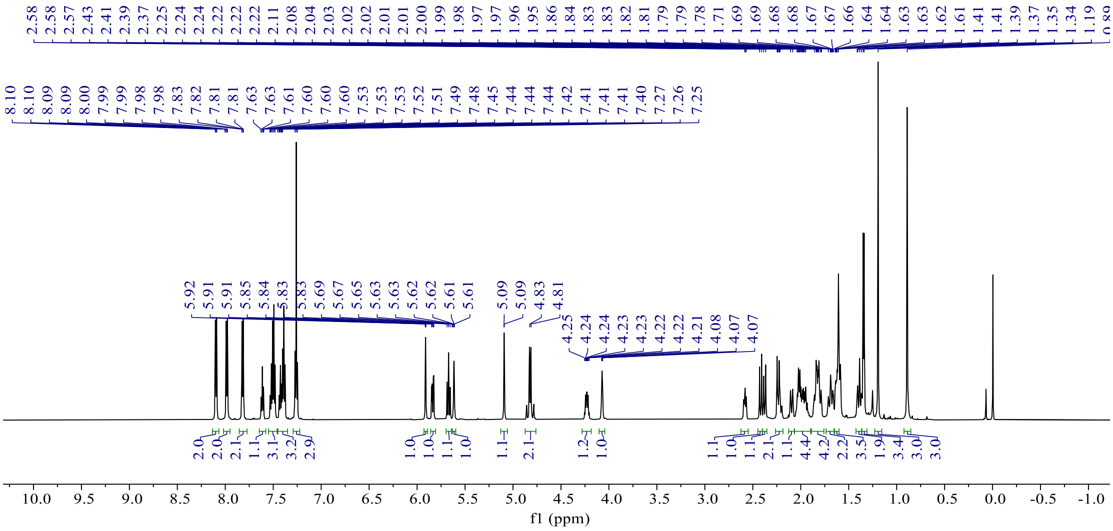

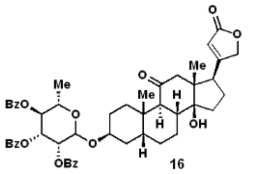

16

^{13}C NMR, $CDCl_3$

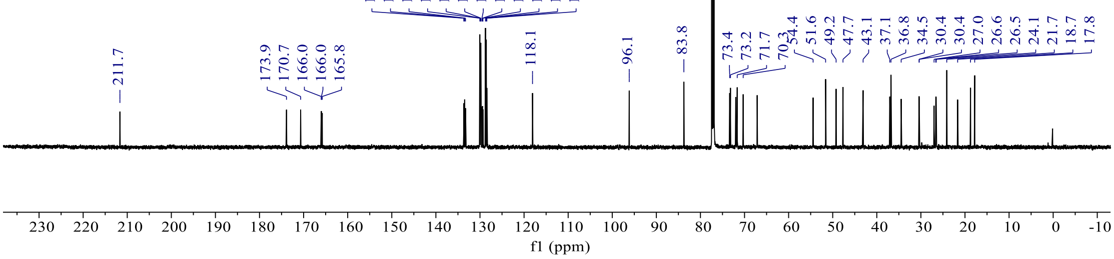

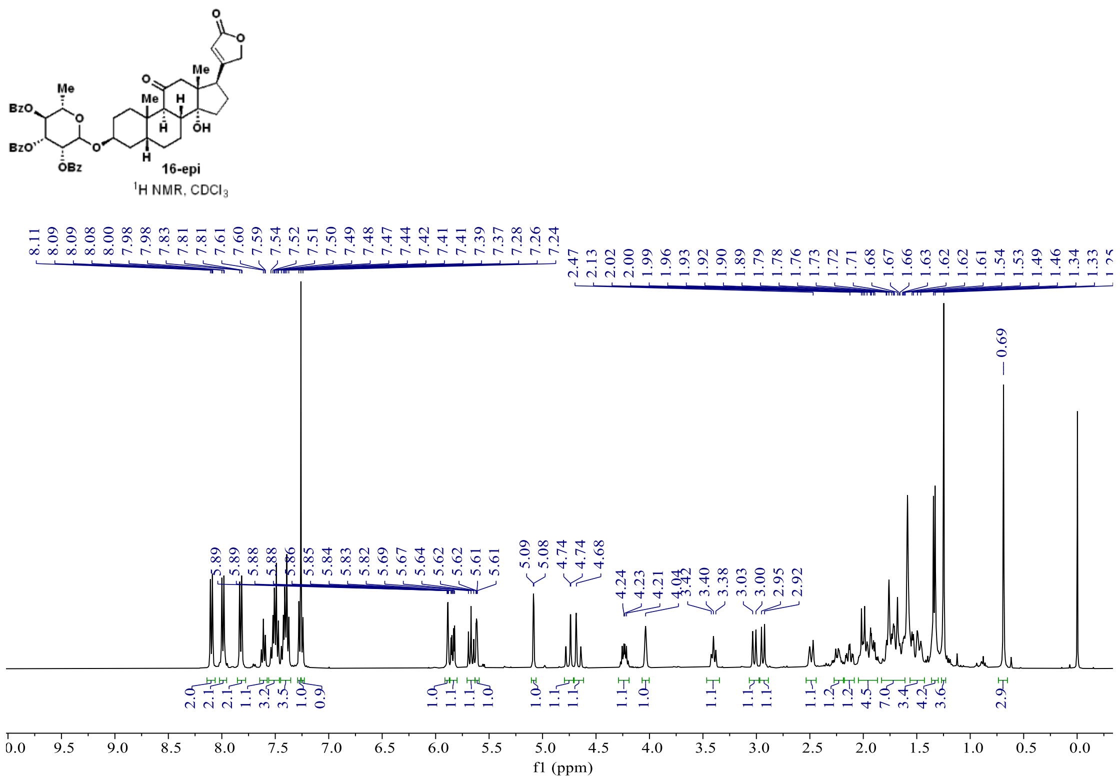
Me
BzO
BzO
OBz
O
Me
Me
H
H
H
OH
O
16-epi
^{1}H NMR, CDCl$_3$
8.11 8.09 8.09 8.08 8.00 7.98 7.98 7.83 7.81 7.81 7.61 7.60 7.59 7.54 7.52 7.51 7.50 7.49 7.48 7.47 7.44 7.42 7.41 7.41 7.39 7.37 7.28 7.26 7.24
2.47 2.13 2.02 2.00 1.99 1.96 1.93 1.92 1.90 1.89 1.79 1.78 1.76 1.73 1.72 1.71 1.68 1.67 1.66 1.63 1.62 1.61 1.54 1.53 1.49 1.46 1.34 1.33
0.69
5.89 5.89 5.88 5.88 5.86 5.85 5.84 5.83 5.82 5.69 5.67 5.64 5.62 5.62 5.61 5.61
5.09 5.08 4.74 4.74 4.68
4.24 4.23 4.21 4.04 3.42 3.40 3.38 3.03 3.00 2.95 2.92
2.0 2.1 2.1 1.1 3.2 3.5 1.0 0.9 1.0 1.1 1.1 1.0 1.0 1.1 1.1 1.1 1.0 1.1 1.1 1.1 1.1 1.2 1.2 4.5 7.0 3.4 4.2 3.6 2.9
f1 (ppm)

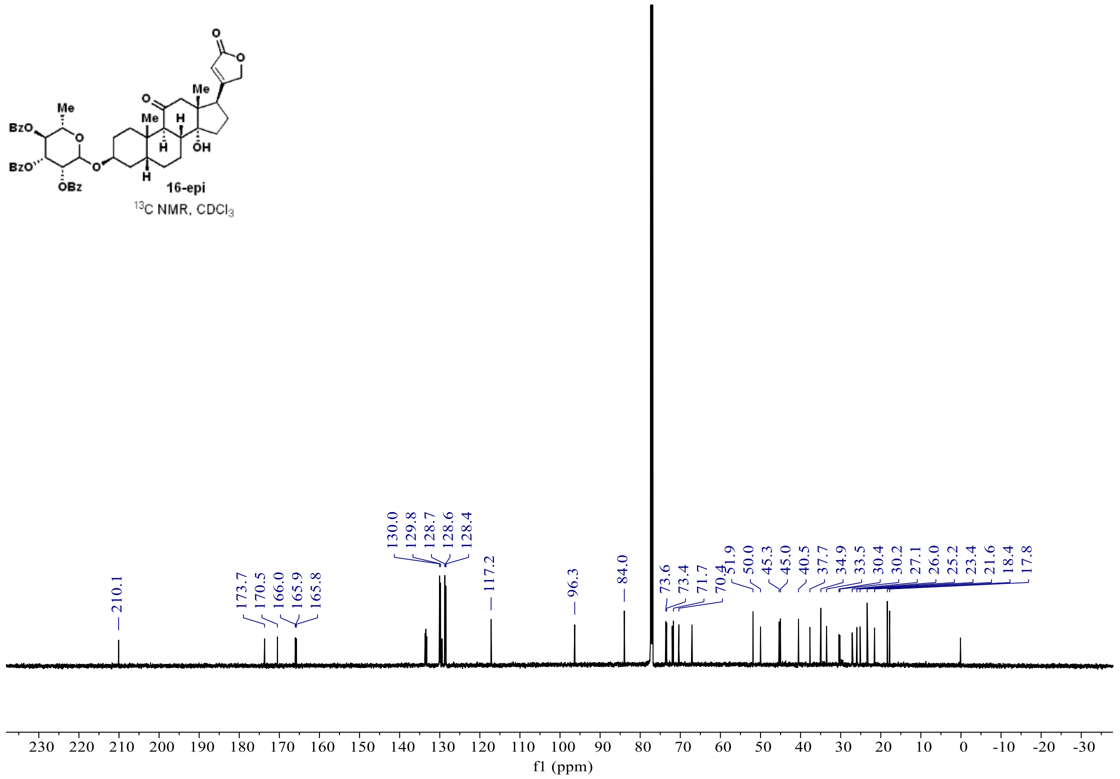
16-epi
^{13}C NMR, CDCl$_3$
210.1
173.7 170.5 166.0 165.9 165.8
130.0 129.8 128.7 128.6 128.4
117.2
96.3
84.0
73.6 73.4 71.7 70.4 51.9 50.0 45.3 45.0 40.5 37.7 34.9 33.5 30.4 30.2 27.1 26.0 25.2 23.4 21.6 18.4 17.8
f1 (ppm)

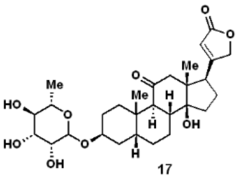

17

^{1}H NMR, CD_3OD

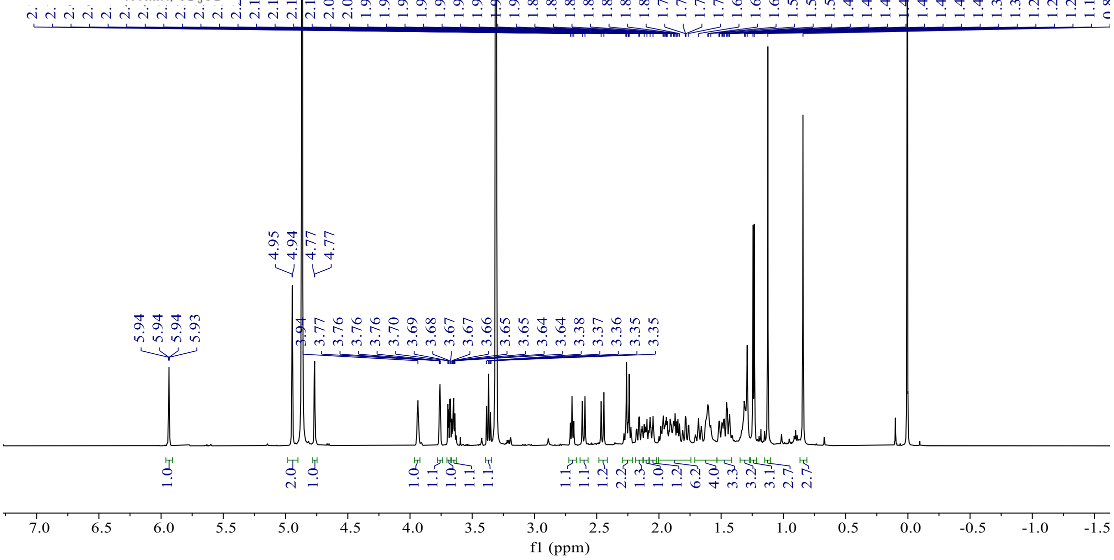

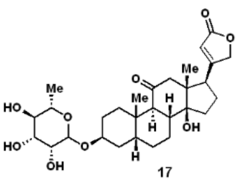

17

^{13}C NMR, CD_3OD

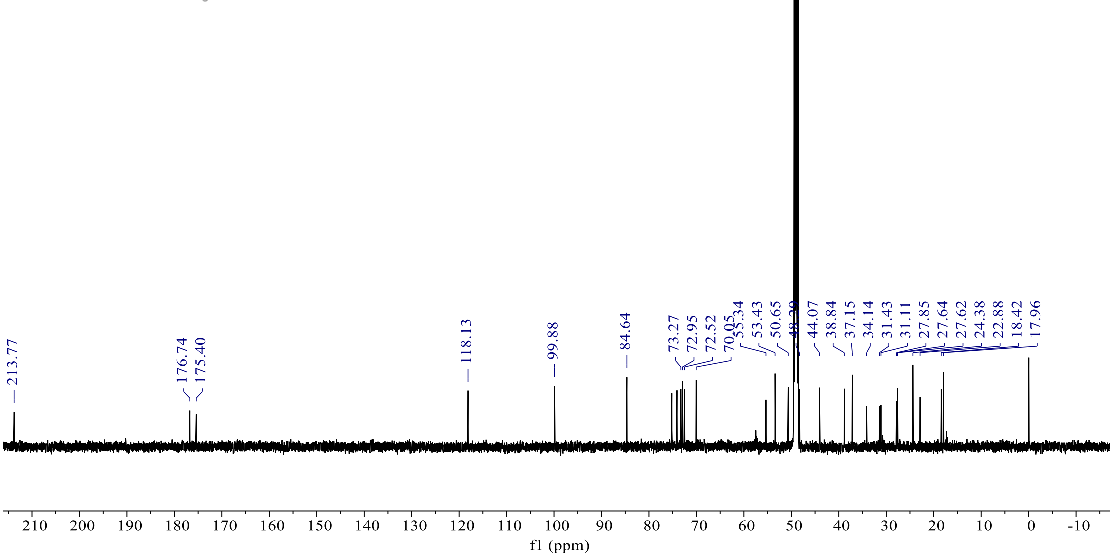

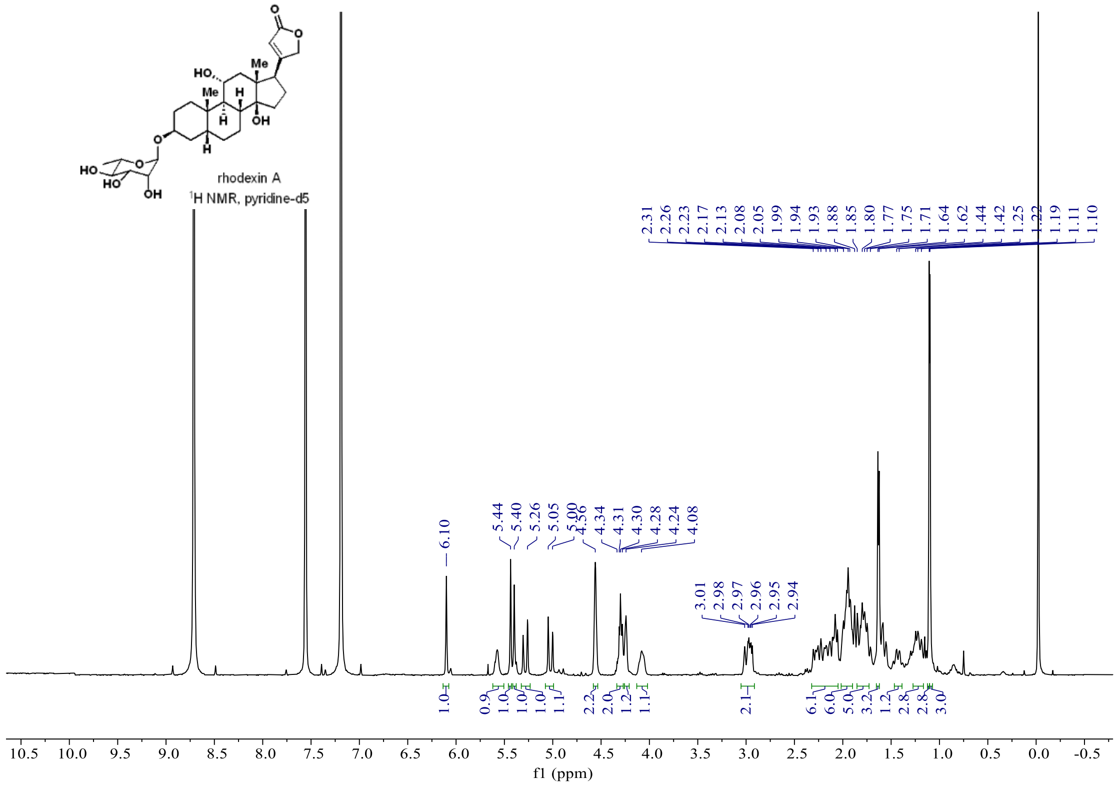
rhodexin A
1H NMR, pyridine-d5
f1 (ppm)

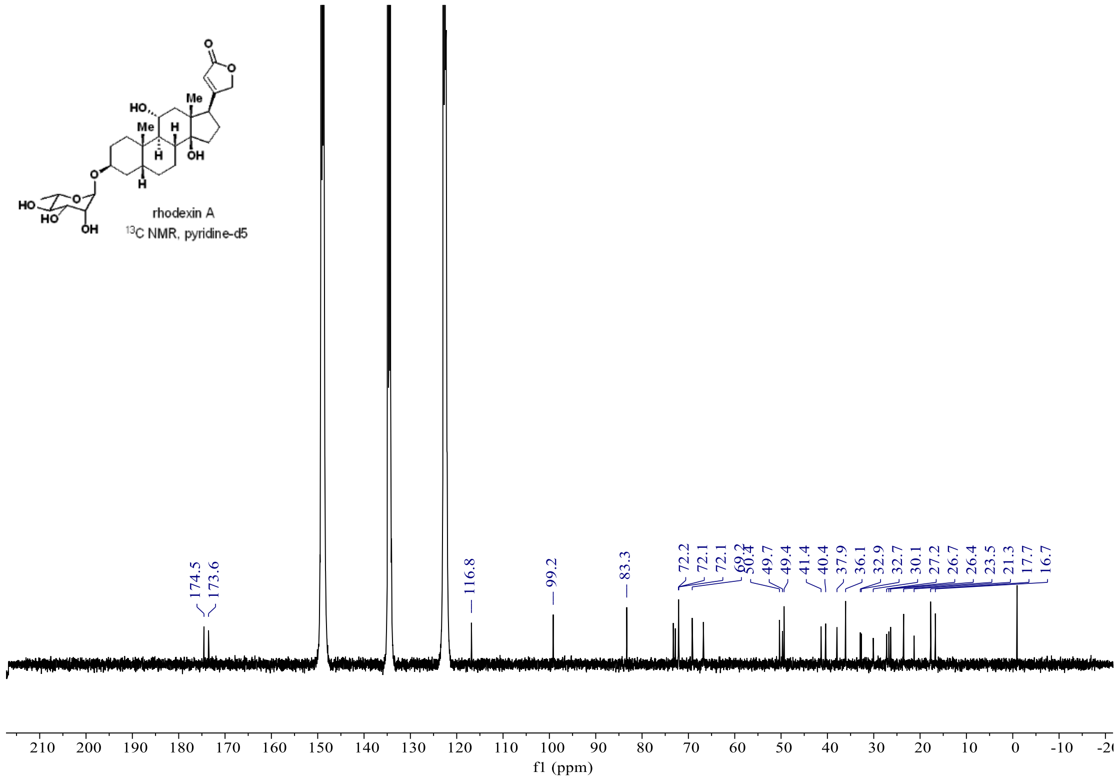
rhodexin A
13C NMR, pyridine-d5
f1 (ppm)
